# Molecular Basis of Rhodomyrtone Resistance in Staphylococcus aureus

**DOI:** 10.1128/mbio.03833-21

**Published:** 2022-02-15

**Authors:** Li Huang, Miki Matsuo, Carlos Calderón, Sook-Ha Fan, Aparna Viswanathan Ammanath, Xiaoqing Fu, Ningna Li, Arif Luqman, Marvin Ullrich, Florian Herrmann, Martin Maier, Anchun Cheng, Fajun Zhang, Filipp Oesterhelt, Michael Lämmerhofer, Friedrich Götz

**Affiliations:** a Microbial Genetics, Interfaculty Institute of Microbiology and Infection Medicine Tübingen (IMIT), University of Tübingen, Tübingen, Germany; b Institute of Pharmaceutical Sciences, University of Tübingen, Tübingen, Germany; c Institute of Organic Chemistry, University of Tübingen, Tübingen, Germany; d Institute of Preventive Veterinary Medicine, College of Veterinary Medicine, Sichuan Agricultural University, Chengdu, China; e Institute of Applied Physics, University of Tübingen, Tübingen, Germany; f Microbial Bioactive Compounds, Interfaculty Institute of Microbiology and Infection Medicine Tübingen (IMIT), University of Tübingen, Tübingen, Germany; g Excellence Cluster 2124 ‘Controlling Microbes to Fight Infections’ (CMFI), University of Tübingen, Tübingen, Germany; Institut Pasteur

**Keywords:** FarR, FarE, isothermal titration calorimetry, lipidomic analysis, P-lipids, rhodomyrtone, Rom, resistance mechanism, *Staphylococcus*, *farR*

## Abstract

Rhodomyrtone (Rom) is a plant-derived broad-spectrum antibiotic active against many Gram-positive pathogens. A single point mutation in the regulatory *farR* gene (farR*) confers resistance to Rom in Staphylococcus aureus (RomR). The mutation in farR* alters the activity of the regulator, FarR*, in such a way that not only its own gene, farR*, but also the divergently transcribed *farE* gene and genes controlled by the global regulator, *agr*, are highly upregulated. Here, we show that mainly the upregulation of the fatty acid efflux pump FarE causes the RomR phenotype, as *farE* deletion in either the parent or the RomR strain (RomR Δ*farE*) yielded hypersensitivity to Rom. Comparative lipidome analysis of the supernatant (exolipidomics) and the pellet fraction revealed that the RomR strain excreted about 10 times more phospholipids (PGs) than the parent strain or the Δ*farE* mutants. Since the PG content in the supernatant (2,244 ng/optical density [OD]) was more than 100-fold higher than that of fatty acids (FA), we assumed that PG interacts with Rom, thereby abrogating its antimicrobial activity. Indeed, by static and dynamic light scattering (SLS and DLS) and isothermal titration calorimetry (ITC) analyses, we could demonstrate that both PG and Rom were vesicular and reacted with each other in milliseconds to form a 1:1.49 [Rom-PG(32:0), where PG(32:0) is PG with C32:0 lipids] complex. The binding is entropically driven and hence hydrophobic and of low specificity in nature. Our results indicate that the cytoplasmic membrane is the actual target of Rom, which is also in agreement with Rom’s induced rapid collapse of the membrane potential and decreased membrane integrity.

## INTRODUCTION

Bacterial infections and particularly antimicrobial resistance to antibiotics are worldwide problems. A crucial point is therefore the development of new antimicrobial strategies against drug-resistant Gram-positive and Gram-negative bacteria that cause acute or chronic infections. While many antibiotics are derived from bacteria and fungi, there is a growing trend of looking more closely to the antimicrobial potential of plant-derived compounds. One such compound is rhodomyrtone (Rom), originally isolated from plant extracts of Rhodomyrtus tomentosa (1). Rom has good antimicrobial activity against a wide range of Gram-positive bacteria, including multidrug-resistant Enterococcus faecalis, Propionibacterium acnes, Staphylococcus aureus, Streptococcus pneumoniae, and Streptococcus pyogenes (2–6). In a mouse model of skin infection with methicillin-resistant Staphylococcus aureus (MRSA), it has been shown that rhodomyrtosone B prevents skin ulcer formation and reduces the incidence of infection-related morbidity; its activity was comparable to that of vancomycin (7). Structural analysis revealed that Rom belongs to the acylphloroglucinol class (1). The elaboration of a chemical synthesis route of Rom and thus the production of larger quantities enabled the study of the mode of action (8, 9).

Rom interferes with none of the classical antibiotic targets, such as peptidoglycan biosynthesis, DNA replication, translation, and transcription, but targets the cell membrane by causing a strong dissipation of the membrane potential and release of ATP and cytoplasmic proteins (10). Rom does not seem to be a typical membrane-inserting molecule, but it disrupts the membrane by inducing the formation of large invaginations and transiently binding to phospholipid (P-lipid) (phosphatidylglycerol [PG]) head groups (11). Interestingly, the antimicrobial activity of Rom could be counteracted by supplementing the medium with certain fatty acids (FAs) (pentadecylic acid, palmitic acid, and stearic acid) (10).

In our previous study, we were able to isolate Rom-resistant mutants by subculturing S. aureus HG001 in medium supplemented with Rom (12) and to attribute Rom resistance to a single point mutation in the coding region of *farR* (regulator of fatty acid resistance). FarR belongs to the TetR family of regulators (TFRs), which are widely associated with antibiotic resistance and the regulation of genes encoding small-molecule exporters, but they are also involved in controlling many other aspects of prokaryotic physiology (13). In S. aureus the divergently transcribed genes *farR-farE*, first described by Alnaseri et al. in 2015 (14), encode the regulator (FarR) and the efflux pump (FarE) that confer resistance to the antimicrobial fatty acids linoleic and arachidonic acids (14).

The Rom-resistant mutant S. aureus HG001 *farR_Cys116Arg_*, henceforth referred to as RomR, exhibited an increase in the MIC from 0.5 μg/mL to >128 μg/mL (12). A comparative transcriptome analysis revealed that many genes were differentially expressed in wild-type S. aureus HG001 and the isogenic RomR mutant, suggesting that the mutant FarR_Cys116Arg_ (FarR*) displays an altered regulatory activity. In particular, *farR*, *farE*, *agr* (accessory gene regulator), and the Agr-controlled virulence genes were upregulated in the RomR mutant, the latter explaining the increased pathogenicity of the RomR mutant (12). We speculated that the upregulation of *farE* is likely to play a crucial role in Rom resistance, as a *farE* deletion mutant strain (Δ*farE*) became hypersensitive to Rom.

In this study, we demonstrated by qualitative and quantitative lipidomic analyses that the RomR strain releases large amounts of PGs into the supernatant and cell envelope, suggesting that FarE acts as a PG efflux pump. Rom resistance is mediated by interaction of PG with Rom, thereby abrogating its antimicrobial activity.

## RESULTS

### Deletion of *farE* in S. aureus HG001 and its isogenic RomR mutant renders the mutants hypersensitive to Rom.

**FIG 1:**
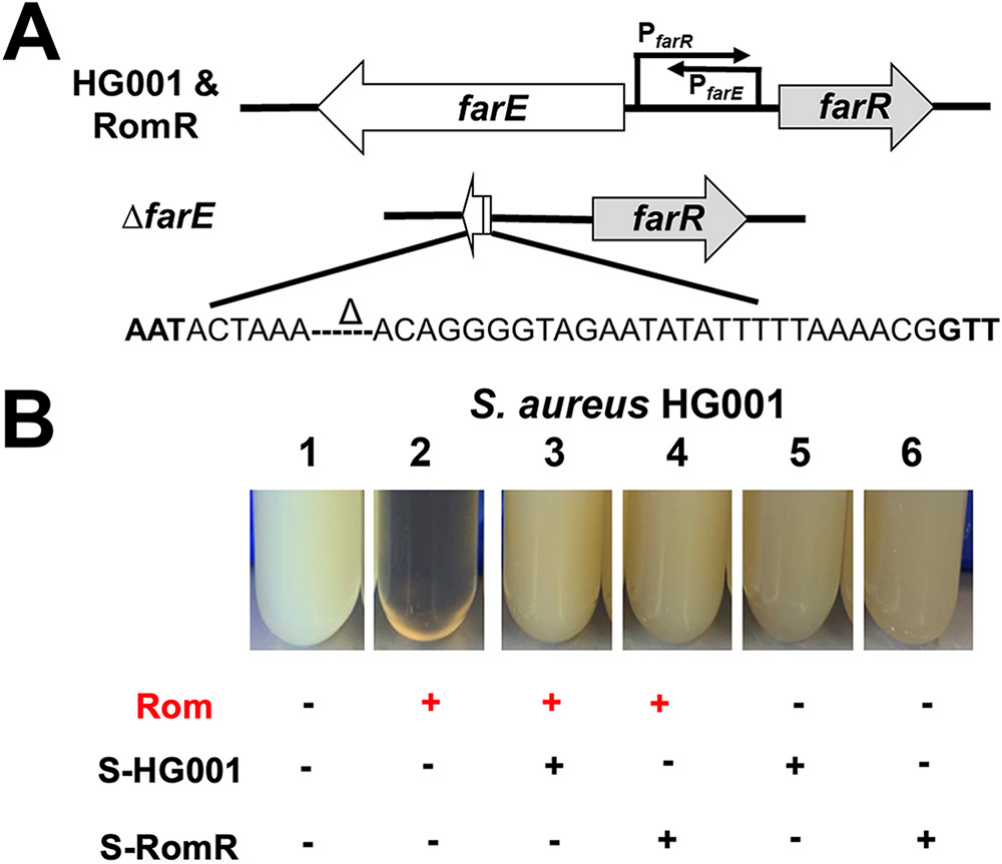
Rom susceptibility and growth of *farE* deletion mutants. (A) Overview of the strategy used to generate the Δ*farE* deletion mutants in HG001 and RomR strains. The delta symbol in the sequence indicates a deleted region. (B) Rom’s antimicrobial activity can be abrogated by addition of culture supernatant of HG001 and the RomR mutant from the end of the exponential phase. Growth of S. aureus HG001 was inhibited by Rom (2 μg/mL) (compare tubes 1 and 2). The addition of 20-times-concentrated supernatant (1:20 [vol/vol]) of HG001 (tube 3) and 2-times-concentrated supernatant (1:2 [vol/vol]) of RomR (tube 4) rescued the growth, while supernatants alone (tubes 5 and 6) had no impact on growth. Cells were grown in BM for 12 h.

To investigate whether *farE* is responsible for Rom resistance in RomR, we constructed markerless *farE* deletion mutants in the S. aureus HG001 wild-type and RomR mutant strains (HG001 Δ*farE* and RomR Δ*farE*, respectively). Since the promoters of *farE* and *farR* are divergent and the corresponding transcripts overlapped as described by the group of Martin McGavin (15), we were cautious not to disrupt any of the promoter regions and constructed an internal deletion in *farE* leaving the first 10 codons and the last codons, including the stop codon TAA, intact (Fig. 1A). Deletion of *farE* led to a decrease in the MIC values even in the RomR mutant, with a drop from >128 to 0.5 μg/mL (Table 1).

**TABLE 1:** MICs of Rom

Strain	MIC (μg/mL)
HG001	1.0
RomR	>128.0
HG001 Δ*farE*	0.5
RomR Δ*farE*	0.5

Next, we examined the impact of Rom on the growth of HG001 and RomR and their respective Δ*farE* mutants. Rom (8 μg/mL) was added to basic medium (BM) 1 h after starting the incubation, and the growth of S. aureus clones was followed for 20 h. Growth of HG001 was inhibited for the first 12 h but resumed thereafter. Growth of HG001 Δ*farE* was inhibited for the whole period. Growth of RomR mutant was unaffected in the presence of Rom (completely resistant to Rom), while growth of RomR Δ*farE* was inhibited for about 15 h and resumed thereafter (see Fig. S1 in the supplemental material). We assume that during the 12-h lag phase, HG001 accumulated sufficient PGs to neutralize Rom, allowing it to grow.

10.1128/mbio.03833-21.1 FIG S1 Impact of Rom on the growth of HG001 and RomR and their respective Δ*farE* mutants. The growth of the S. aureus clones in BM supplemented with Rom (8 μg/mL) was followed for 24 h. Growth of HG001 was inhibited for about 12 h but resumed thereafter; growth of HG001 Δ*farE* was inhibited for the whole period. Growth of the RomR mutant was unaffected in the presence of Rom (completely resistant to Rom), while the growth of RomR Δ*farE* was inhibited for about 15 h and resumed thereafter. The result indicate that FarE is crucial for Rom resistance. The graph is a representative of one of the triplicate growth studies performed. Download FIG S1, PDF file, 0.2 MB. Copyright © 2022 Huang et al. 2022 Huang et al. https://creativecommons.org/licenses/by/4.0/ This content is distributed under the terms of the Creative Commons Attribution 4.0 International license.

Furthermore, we investigated the effect of Rom on the killing of HG001 and RomR and their respective Δ*farE* mutants. The killing of the S. aureus clones in BM supplemented with Rom (8 μg/mL) was followed for 8 h. HG001 and HG001 Δ*farE* were almost completely killed after 7 to 8 h, while the growth of the RomR mutant continued. With RomR Δ*farE*, mutant killing was also observed, but it was markedly delayed compared to the case with HG001 and HG001 Δ*farE* (Fig. S2). Together, these results show that deletion of *farE* in both HG001 and RomR rendered the strains hypersensitive to Rom, indicating that FarE is crucial for Rom resistance. We also observed that in RomR Δ*farE*, the killing rate was lower and the strain even started to regrow after 7 h, suggesting that FarR* regulates some other genes apart from *farE* that contribute to the revival of the mutant.

10.1128/mbio.03833-21.2 FIG S2 Impact of Rom on the killing of HG001 and RomR and their respective Δ*farE* mutants. The killing of the S. aureus clones in BM supplemented with Rom (8 μg/mL) was followed for 8 h. Cells were inoculated to a concentration of about 6 × 10^5^ CFU/mL. HG001 and HG001Δ*farE* were almost completely killed after 7 to 8 h, while growth of the RomR mutant continued. With the RomR Δ*farE* mutant, killing was also observed, but it was markedly delayed compared to that of HG001 and HG001 Δ*farE*. Each bar represents the mean ± SD from three independent biological replicates. Download FIG S2, PDF file, 0.1 MB. Copyright © 2022 Huang et al. 2022 Huang et al. https://creativecommons.org/licenses/by/4.0/ This content is distributed under the terms of the Creative Commons Attribution 4.0 International license.

### Concentrated supernatant of HG001 and RomR can neutralize Rom’s antimicrobial activity.

One of our main questions was whether Rom resistance was due to Rom being expelled from the cell by the efflux pump FarE or whether the excreted PGs and fatty acids neutralized Rom. While two-times-concentrated supernatant of RomR strains grown to the end of the exponential phase was already able to protect HG001 from Rom, the supernatant of HG001 needed to be concentrated 20 times to achieve similar protection (Fig. 1B). This suggests that the substances leading to Rom resistance are the same in the parental strain and the RomR mutant but that their concentrations are different. Since we have previously shown that certain fatty acids can partially abolish the antibiotic activity of Rom (12), we assumed that FarE secreted FAs and lipids to neutralize Rom’s activity. To verify this hypothesis, we carried out qualitative and quantitative lipid and FA analyses of the supernatant of parent strain HG001 as well as RomR and Δ*farE* mutants.

### Determination of excreted lipids/FAs in the supernatant and pellet wash of HG001 and its mutants.

For lipidomic analysis of secreted as well as cell-bound lipids/FAs, we used a chemically defined minimal medium (38) which is free of fatty acids and lipids. When grown in BM, HG001, RomR, HG001 Δ*farE*, and RomR Δ*farE* showed no discernible differences. However, their growth in the defined minimal medium was generally decreased and growth of the two Δ*farE* mutants was somewhat delayed (Fig. S3). The cultures were harvested when they reached the end of the exponential growth phase (optical density at 578 nm [OD_578_] = 0.9 to 1.0). For the calculation of the sample concentration and the structure, internal standards (ISs) as well as the TripleTOF system and liquid chromatography-electrospray ionization-tandem mass spectrometry (LC-ESI-MS/MS) were used. We analyzed not only the lipid/FAs in the culture supernatant but also those that were loosely bound to the cell wall, referred to as “pellet wash,” by detaching them from the cell wall with 90% isopropanol treatment. Absolute amounts of FAs/lipids were calculated in nanograms/OD_578_ and adjusted to the corresponding OD_578_ of 1.0. As described earlier, S. aureus synthesizes mainly saturated FAs (16), and in this study, too, we detected only saturated FAs in free or lipid-bound form, suggesting that unsaturated FAs were not synthesized.

10.1128/mbio.03833-21.3 FIG S3 Comparative growth of HG001 and its mutants in defined minimal medium using a microplate reader. Each point in the graph is the mean ± SD of three independent biological replicates; bar indicates the end of the exponential phase (OD_578_ ≈ 0.9), at which the cells were harvested for lipidomic analysis. Download FIG S3, PDF file, 0.1 MB. Copyright © 2022 Huang et al. 2022 Huang et al. https://creativecommons.org/licenses/by/4.0/ This content is distributed under the terms of the Creative Commons Attribution 4.0 International license.

### Free FAs.

**FIG 2:**
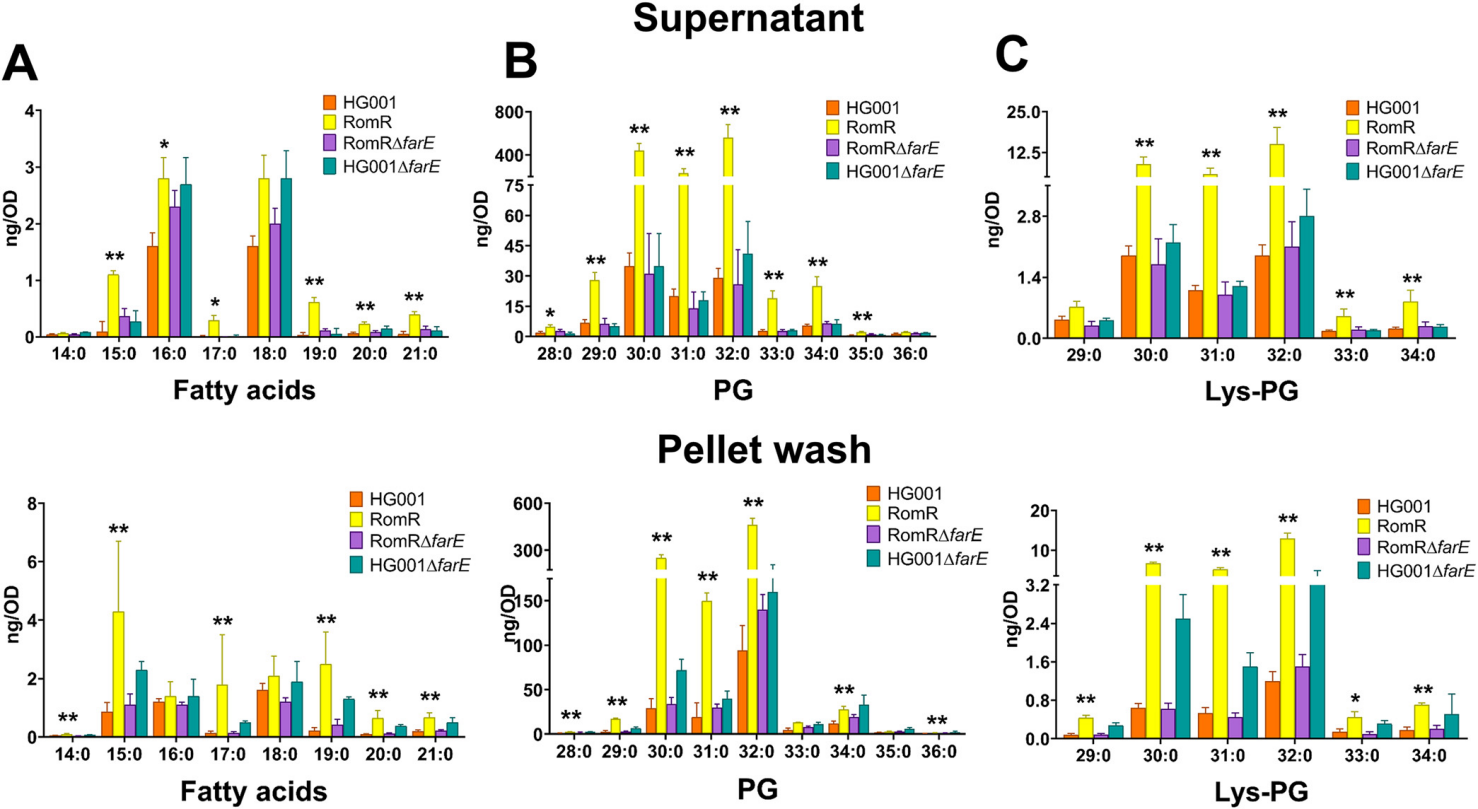
Lipidomic analysis in supernatants (upper row) and pellet wash (lower row) of HG001, RomR, HG001 Δ*farE*, and RomR Δ*farE*. The bar charts show the amounts of FAs ranging from C14:0 to C21:0 (A), phosphatidylglycerols (PGs) ranging from C28:0 to C36:0 (B), and lysyl phosphatidylglycerol (Lys-PG) (C) ranging from C29:0 to C34:0. Absolute amounts of FAs and lipids were calculated in nanograms per OD_578_ of the bacterial cultures grown until the end of the exponential phase and adjusted to the corresponding OD_578_ of 1.0. The supernatant and pellet were separated by centrifugation for lipidomic analyses. The pellet wash was obtained by treating the pellets with 90% isopropanol to remove the fatty acids and lipids from the surface of cells. Each bar represents the mean ± SD from five independent biological replicates. *P* values were obtained using the Mann-Whitney U test for the comparison between HG001 and RomR, with *P* values of <0.05 and <0.01 shown with “*” and “**,” respectively.

Exolipidomic analyses of the supernatant and pellet wash showed that there were no large differences in the majority of even-numbered FAs (C16:0 and C18:0) between HG001 and the mutants. However, the RomR mutant excreted about 2-fold more odd-numbered FAs (C15:0, C17:0, C19:0, and C21:0) than HG001 and the two Δ*farE* mutants (Fig. 2A). If we add up all the FAs in the supernatant and pellet wash, the following values were achieved: 22 ng/OD for RomR, 8 ng/OD for HG001, 9 ng/OD for RomR Δ*farE*, and 15 ng/OD for HG001 Δ*farE*. The distributions of FAs in supernatant and pellet wash were roughly comparable, with only approximately 4 times more C15:0 in the pellet wash than in the supernatant. Since there was only a 4-fold difference in secreted FAs between the parental strain and the RomR mutant, we hypothesized that secreted lipids might be responsible for the Rom resistance in RomR.

### PG.

Phosphatidylglycerol (PG) was the most excreted lipid in terms of quantity, and it is in the levels of PG that we observed the greatest difference between RomR and HG001 and the Δ*farE* mutants. On average, the RomR strain released about 8 to 10 times more lipids than HG001 and the Δ*farE* mutants. The most abundant lipids ranged from C29:0 to C34:0, with a peak from C30:0 to C32:0 (Fig. 2B). If we add up all the lipid structures in the supernatant and pellet wash, we come to about 2,244 ng/OD for RomR, 268 ng/OD for HG001, 332 ng/OD for RomR Δ*farE*, and 448 ng/OD for HG001 Δ*farE*.

### Lys-PG.

With respect to the distribution of the chain length of the esterified FAs, we observed a pattern similar to that of PG except for the amount of released lysyl phosphatidylglycerol (Lys-PG), which was about 30-fold lower than for PG (Fig. 2C). If we add up all the Lys-PG structures in the supernatant and pellet wash, we come to about 59 ng/OD for RomR, 8 ng/OD for HG001, 9 ng/OD for RomR Δ*farE*, and 16 ng/OD for HG001 Δ*farE*. It is reasonable that only a small amount (about 3%) of the PG is lysylated, since expression of MprF (multiple peptide resistance factor) is inducible (17) and cells were not grown under conditions that are optimal for MprF expression or harvested at an optimal time point for MprF expression.

### DG and MGDG.

**FIG 3:**
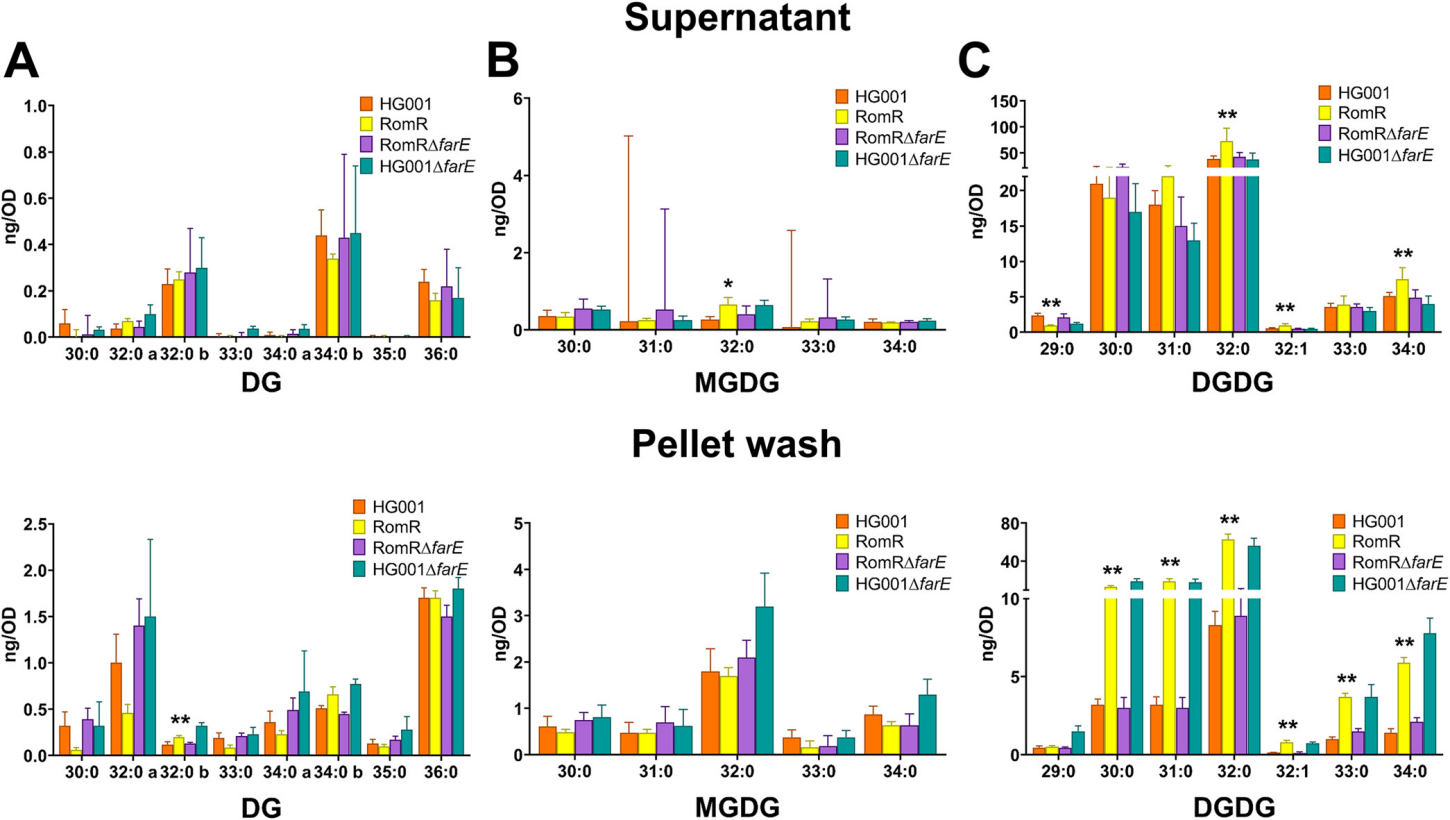
Lipidomic analysis of diacylglycerol (DG), monogalactosyldiacylglycerol (MGDG), and digalactosyldiacylglycerol (DGDG) in supernatants (upper row) and pellet wash (lower row). The bar charts show the amounts of DGs ranging from 30:0 to 36:0 (A), MGDGs ranging from 30:0 to 34:0 (B), and DGDGs ranging from 29:0 to 34:0 (C). *P* values were obtained using the Mann-Whitney U test for the comparison between HG001 and RomR, with *P* values of <0.05 and <0.01 shown with “*” and “**,” respectively.

Only trace amounts of diacylglycerol (DG) and monogalactosyldiacylglycerol (MGDG) were released by all strains, and there was no remarkable difference between RomR and HG001 or the Δ*farE* mutants (Fig. 3). The total amount of DG in the supernatant per strain was on the order of about 1 ng/OD, and that in the pellet wash was about 5 ng/OD (Fig. 3A); the total amounts of MGDG were on the same order, i.e.,1 to 2 ng/OD in the supernatant and about 5 to 8 ng/OD in the pellet wash (Fig. 3B). These results indicate that FarE does not really contribute to the release of DG or MGDG.

### DGDG.

Digalactosyldiacylglycerol (DGDG) was found in substantial levels in the supernatant, amounting to about 110 ng/OD for each strain, and there were no significant differences among the four strains, except for HG001 and RomR (Fig. 3C). Only in the pellet fraction did we see an approximately 4-fold-larger amount of DGDG in RomR than in the other strains.

Summarizing the lipidomic results, we can say that the RomR strain excreted multiple amounts of PG and Lys-PG, compared to the other strains (Table 2). By far the most abundant lipid structure released was PG, followed by Lys-PG and FAs. DG and MDG were released in only tiny amounts, and we found no indication for a higher release in the RomR strain. DGDG was released in larger amounts than DG and MDG, but only in the pellet wash did we observe a 2-times-higher content in the RomR strain than in the others. The overexpression of FarE in the RomR mutant (12) led the mutant to become a hyperreleaser of PG. Deletion of the *farE* gene in the RomR mutant reversed the phenotype. This appears to represent direct evidence that overexpression of FarE is responsible for release of PGs.

**TABLE 2:** Contents of FAs, lipids, PGs, sugar lipids in supernatant of Staphylococcus aureus

Compound(s)	Most abundant FAs (from high to low)	Total amt (ng/OD)	
		HG001	RomR
FAs	C18:0, C16:0, C15:0, C19, C21:0, C20:0, C17:0, C14:0	8	22
PG	C32:0, C30:0, C31:0, C34:0, C29:0, C33:0, C28:0, C35:0, C36:0	268	2,244
Lys-PG	C32:0, C30:0, C31:0, C29:0, C34:0, C33:0	8	59
DG	C36:0, C32:0a, C34:0b	5	4
MGDG	C32:0, C34:0, C30:0, C31:0, C33:0	5	5
DGDG	C32:0, C30:0, C31:0, C34:0, C33:0, C29:0, C32:1, C33:1	107	233

### Rom does not induce the release of fatty acids or PG.

To investigate the effects of Rom treatment on the release of lipids by the parent strain HG001 and the mutant strain RomR (Fig. S4), Rom was added at a sublethal concentration (0.3 μg/mL) at early growth phase, and samples were harvested and processed as described above for untreated cultures. The lipidomic analysis showed essentially the same pattern as for the untreated samples. There was no significant difference in the release of FAs, PG, and Lys-PG in the presence of Rom (Fig. S4), indicating that Rom does not induce the release of these compounds in S. aureus.

10.1128/mbio.03833-21.4 FIG S4 Effect of subinhibitory concentrations of Rom on the release of fatty acids and lipids by S. aureus HG001 and RomR strains in the supernatant (top) and pellet wash (bottom). The bar charts show the amount of fatty acids ranging from C14:0 to C21:0 (A), phosphatidylglycerols (PG) ranging from C28:0 to C36:0 (B), and lysyl phosphatidylglycerols (Lys-PG) ranging from C29:0 to C:34 (C) detected in both strains in the presence of subinhibitory concentration of Rom (0.3 μg/mL). All absolute amounts of FAs and lipids are reported in nanograms/OD_578_ of the bacterial cultures grown until the end of the exponential phase and adjusted to the corresponding OD_578_ of 1.0. The supernatant and pellet were separated by centrifugation for lipidomic analyses. The pellet wash was obtained by treating the pellets with 90% isopropanol to remove the fatty acids and lipids from the surface of cells. Each bar shown in the chart represents the mean ± SD from five independent biological replicates. Download FIG S4, PDF file, 0.2 MB. Copyright © 2022 Huang et al. 2022 Huang et al. https://creativecommons.org/licenses/by/4.0/ This content is distributed under the terms of the Creative Commons Attribution 4.0 International license.

### Certain FAs and PGs can neutralize the activity of Rom.

**FIG 4:**
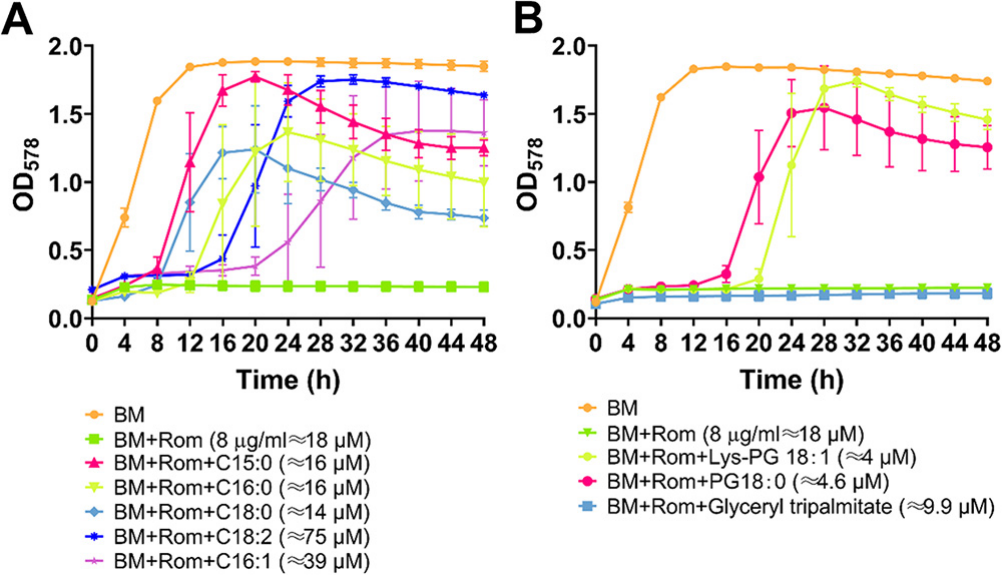
Impact of exogenous supplementation of fatty acids and phospholipids on the activity of Rom. S. aureus HG001 was grown in BM with Rom (8 μg/mL) and various supplementations. For panel A, supplementations included the following FAs: 4 μg/mL of C15:0 (≈16 μM), 4 μg/mL of C18:0 (≈14 μM), 4 μg/mL of C16:0 (≈16 μM), 10 μg/mL of C16:1 (≈39 μM), and 21 μg/mL of C18:2 (≈75 μM). For panel B, supplementations included 8 μg/mL of the lipid glyceryl tripalmitate (≈9.9 μM) and phospholipids as follows: 4 μg/mL of PG 18:0 (≈4.6 μM) and 4 μg/mL of Lys-PG 18:1 (≈4 μM). Wells containing tryptic soy broth (TSB) only and bacteria with Rom (8 μg/mL) alone were included as negative and positive controls, respectively. Growth of the bacteria was measured at OD_578_ every 4 h for 48 h using a Varioskan Lux microplate reader (Thermo Scientific) in a 48-well plate. Each point in the graph is the mean ± SD from three independent biological replicates.

Next, we investigated which of the lipid components exported by FarE can most effectively neutralize the antimicrobial activity of Rom. For this, we supplied the medium (BM) with Rom (8 μg/mL ≈ 18 μM) and different lipid components in approximately the same molarity. Rom alone completely inhibited growth (Fig. 4). Among the FAs tested, C15:0 was the most efficient in rescuing growth in the presence of Rom; however, the onset of growth was delayed by 8 h. Unsaturated FAs like C18:2 and C16:1 had a much smaller effect than C15:0 (Fig. 4A). The P-lipids PG(18:0) [where PG(18:0) is PG with C18:0 lipids] and Lys-PG(18:1) also could abrogate Rom’s antimicrobial activity but only after a lag phase of 16 to 20 h (Fig. 4B). Interestingly, triacylglyceride showed no effect. As a control, we also tested whether exogenous supplementation of FAs/lipids/PGs had an effect on growth of HG001. However, neither the tested FAs nor Lys-PG or PG affected growth (Fig. S5A and B).

10.1128/mbio.03833-21.5 FIG S5 Impact of exogenous supplementation of FAs/lipids on the growth of HG001 and construction of RomR Δ*mprF*. (A and B) Growth of S. aureus HG001 in BM with supplementations of fatty acids at 4 μg/mL of C15:0 (≈16 μM), 4 μg/mL of C18:0 (≈14 μM), 4 μg/mL of C16:0 (≈16 μM), 10 μg/mL of C16:1 (≈39 μM), and 21 μg/mL of C18:2 (≈75 μM) (A) and lipids at 4 μg/mL of PG(18:0) (≈4.6 μM) and 4 μg/mL of Lys-PG(18:1) (≈4 μM) (B). The well containing BM only and bacteria was regarded as a positive control. Growth of the bacteria was measured by OD_578_ every 4 h for 48 h using a Varioskan Lux microplate reader (Thermo Scientific) in a 48-well plate. Each point in the graph is the mean ± SD from three independent biological replicates. (C) Illustration of RomR Δ*mprF* deletion mutant. Download FIG S5, PDF file, 0.3 MB. Copyright © 2022 Huang et al. 2022 Huang et al. https://creativecommons.org/licenses/by/4.0/ This content is distributed under the terms of the Creative Commons Attribution 4.0 International license.

### MprF has no effect on Rom resistance.

MprF encodes a bifunctional membrane protein that synthesizes the positively charged lipid Lys-PG and subsequently translocates it from the inner to the outer membrane leaflet (18). Therefore, MprF serves as bacterial resistance factor protecting MRSA from cationic antimicrobial peptides (CAMPs) and the lipopeptide antibiotic daptomycin (18). To investigate whether the biosynthesis of Lys-PG plays a role in Rom resistance, we deleted *mprF* in the RomR strain, generating the strain RomR Δ*mprF* (Fig. S5C). However, when we treated the RomR strain and its Δ*mprF* mutant with Rom, we observed no difference in the MIC values of the two strains (both still >128 μg/mL), suggesting that *mprF* does not play a role in Rom resistance.

In summary, our results show that essentially only the compounds most abundantly released by the RomR strain (PGs) can neutralize the activity of Rom. To demonstrate the postulated interaction of Rom with PGs, we used two methods: static and dynamic light scattering (SLS/DLS) and isothermal titration calorimetry (ITC).

### SLS/DLS indicate that Rom interacts with phospholipid PG(32:0).

DLS is a frequently used technique for measuring the size distribution of dispersed particles in the range of 1 to 3,000 nm. For studies of interaction of Rom with P-lipids, we have chosen PG(32:0), 1,2-dipalmitoyl-*sn*-glycero-3-phospho-(1′-rac-glycerol) (sodium salt), as a model PG that is also produced by S. aureus. Both Rom and PG(32:0) are almost insoluble in water. Therefore, we needed to find a common solvent for both compounds. We overcame the problem by dissolving PG(32:0) first in a chloroform/methanol/water mixture, and then we prepared vesicles formed in 10% dimethyl sulfoxide (DMSO)/phosphate-buffered saline (PBS) (pH 7.2), which is the same solvent as used for Rom.

**FIG 5:**
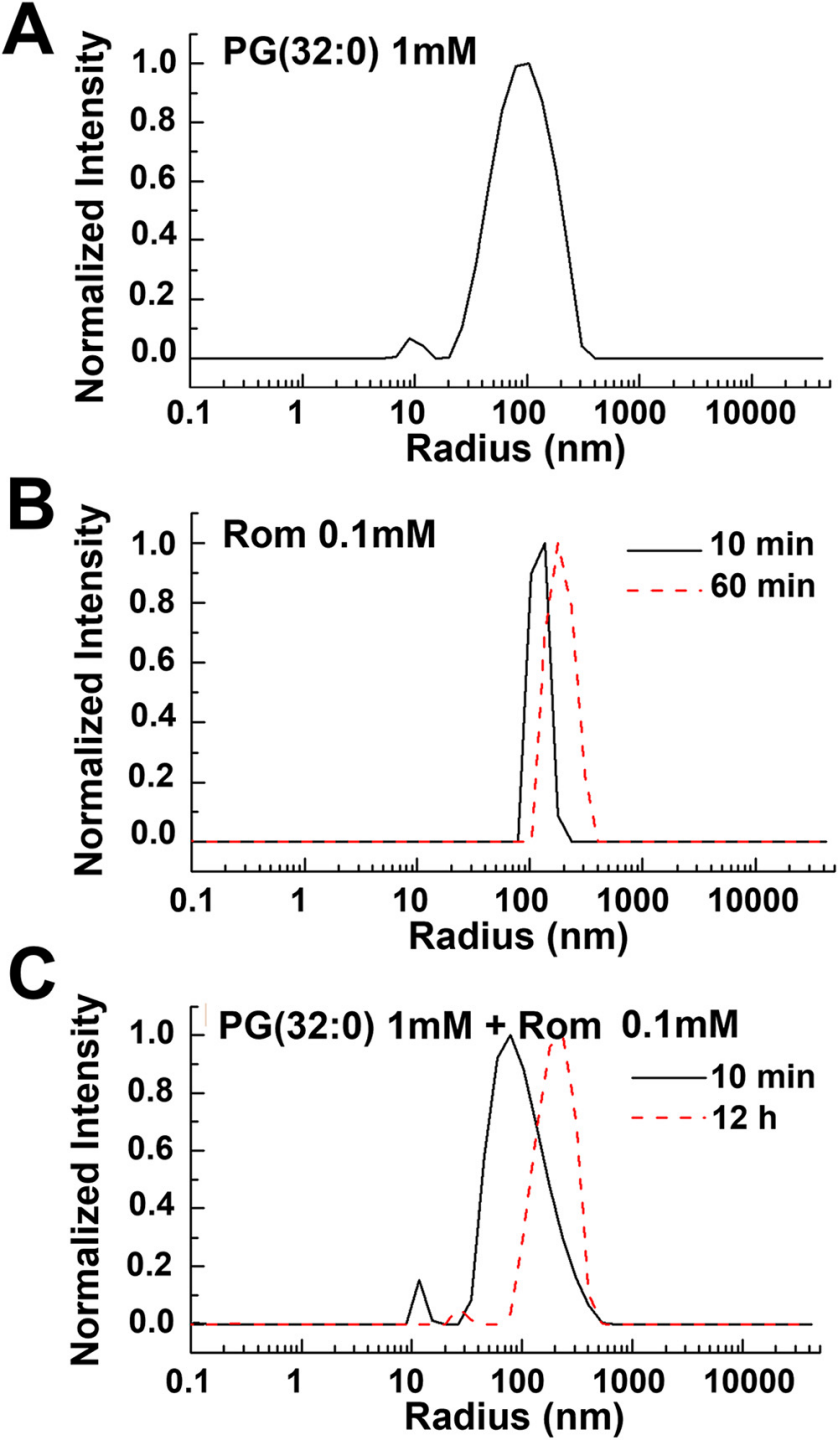
Static and dynamics light scattering (SLS/DLS) of PG(32:0), Rom, and mixed compounds. (A) DLS results for PG(32:0) alone. PG(32:0) at 1 mM was diluted from a 5 mM sample solution. The mean size determined at 90° was 90.8 nm. Further study demonstrated that the size of PG(32:0) vesicles did not change much over time when the concentration was below a certain threshold. (B) DLS results for Rom (0.1 mM) solution. The mean size of nanoclusters was about 120 nm for the freshly prepared sample. The 2nd measurement after 60 min showed an increase in size to 190 nm. For long-time incubation (overnight), visible Rom aggregates appeared. This result suggests that Rom forms nanoclusters after preparation that slowly agglomerate into large aggregates. (C) DLS results for Rom and PG mixture. Rom (0.1 mM) and PG(32:0) (1.0 mM) were mixed in a volume ratio of 1:1. The 1st measurement yielded a mean size of 100 nm, similar to the original size shown in Fig. 1. The 2nd measurement, after overnight incubation, yielded a mean size of about 200 nm, nearly double the size. This result demonstrates that Rom indeed interacts with PG vesicles, leading to an increase of vesicle size.

DLS analysis with 1 mM PG(32:0) (diluted from a 5 mM sample solution) showed that the mean size at a 90° angle was 90.8 nm (Fig. 5A). This size did not change much over time, meaning that PG(32:0) was present as a vesicle under these conditions. Next, we analyzed 0.1 mM Rom solution from a freshly prepared sample and after a 1-h interval. Rom formed nanoclusters with a mean size of 120 nm for the freshly prepared sample. The 2nd measurement after 60 min showed an increase of size to 190 nm (Fig. 5B). After a long incubation (overnight), visible Rom aggregates appeared. These results suggest that Rom forms nanoclusters after preparation and then slowly agglomerates into large aggregates. Finally, we analyzed a mixture of Rom (0.1 mM) and PG(32:0) (1.0 mM) with a volume ratio of 1:1. After 10 min of treatment, the mixture sample was filtered with a 1.6-μm filter. The 1st measurement yielded a mean size of 100 nm (Fig. 5C). The 2nd measurement after an overnight incubation yielded a mean size of about 200 nm, which is double the size. This result is a first hint that Rom indeed interacts with PG(32:0) vesicles, leading to an increase of vesicle size.

### Confirmation of the interaction of Rom with PG(32:0) by ITC.

**FIG 6:**
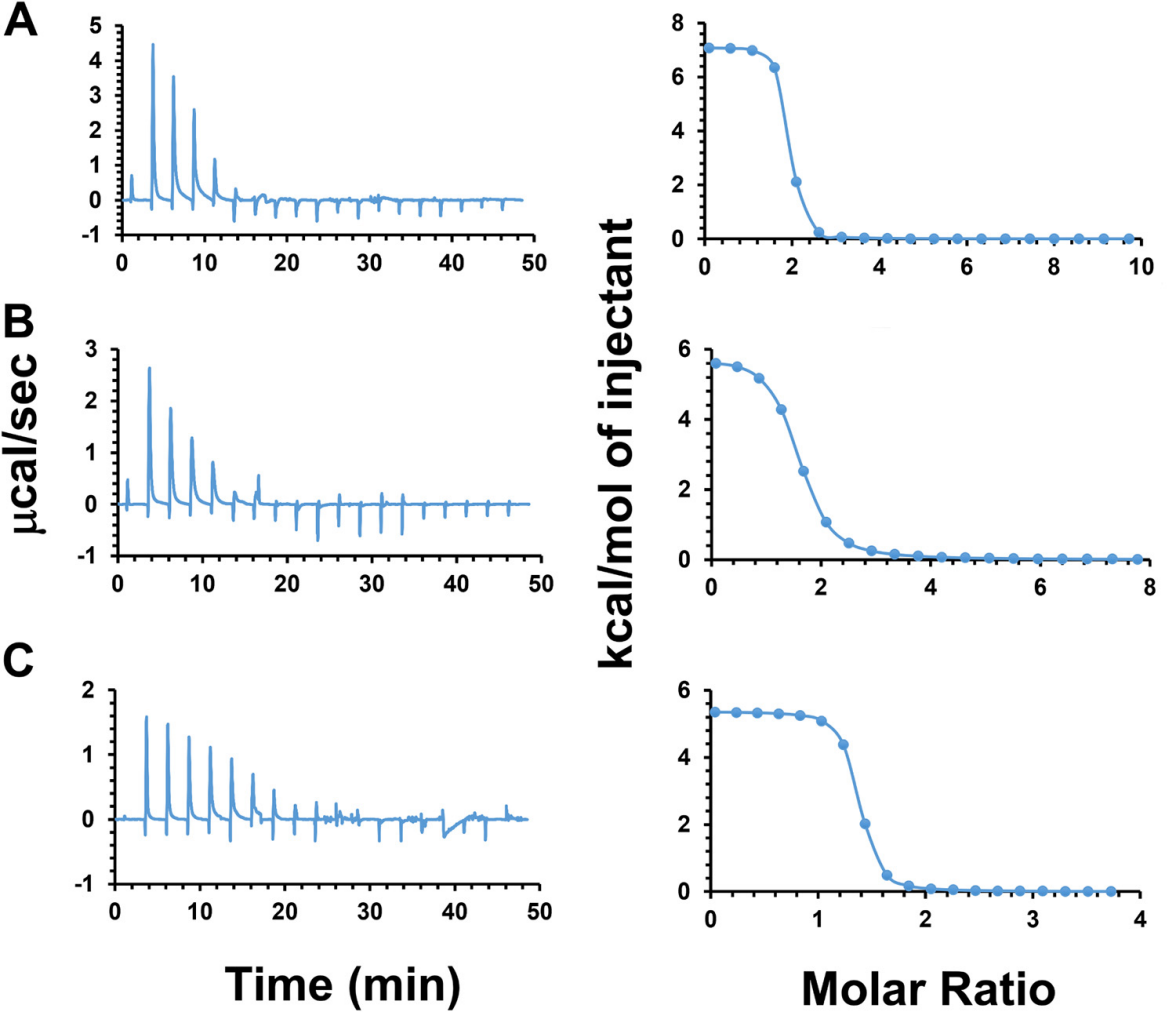
Isothermal titration calorimetry (ITC) of Rom with titrated PG(32:0). Panels A, B, and C present results from three independent experiments with slight changes of injection volume and PG(32:0) concentration. The left side shows the heat change with time. (A) Injection of 2-μL aliquots of 5 mM PG(32:0) into 100 μM Rom. (B) Injection of 2-μL aliquots of 4 mM PG(32:0) into 100 μM Rom. (C) Injection of 1-μL aliquots of 4 mM PG(32:0) into 100 μM Rom. The right side shows the enthalpy change of the binding for each condition. The free energy and entropy were calculated with software provided by Malvern.

To gain further evidence for neutralization of Rom’s activity by the released PGs into the medium, we determined the enthalpy change (Δ*H*), equilibrium binding constant (*K_D_*), and stoichiometry of the reaction (*n*) involved in the interaction between Rom and PG(32:0) using ITC. The binding of Rom and PG(32:0) was determined titrating PG(32:0) into Rom solution at pH 7.2 in 10% DMSO/PBS buffer at 37°C. Three experiments were conducted with different conditions, all of which resulted in similar heat flows (Fig. 6): (i) injection of 2-μL aliquots of 5 mM PG(32:0) into 100 μM Rom (Fig. 6A), (ii) injection of 2-μL aliquots of 4 mM PG(32:0) into 100 μM Rom (Fig. 6B), and (iii) injection of 1-μL aliquots of 4 mM PG(32:0) into 100 μM Rom (Fig. 6C). The interaction was found to be endothermic, as the titration peaks showed the upward position and the corresponding integrated heat was positive (Δ*H* > 0). The contribution of the entropy change (Δ*S*) to the binding was favorable and large (−*T*Δ*S* < 0, where *T* is absolute temperature). In other words, binding is entropically driven and hence hydrophobic and of low specificity in nature. The *n* and *K_D_* were 1.49 (±0.0478) and 2.30 (±0.747) μM, respectively. The interaction of Rom with PG(32:0) was fast and occurred within milliseconds. The data are summarized in Table 3.

**TABLE 3:** Thermodynamic parameters obtained by ITC for Rom binding to PG(32:0) vesicles^*a*^

Expt	System			*n*, sites	*K_D_* (mM)	Δ*H* (kcal/mol)	Δ*G* (kcal/mol)	(−T)Δ*S* (kcal/mol)
	Syringe	Cell	Injection vol					
1	PG, 5 mM	Rom, 100 μM	2 μL	1.72 (±0.048)	0.743 (±0.656)	7.09	−8.7	−15.8
2	PG, 4 mM	Rom, 100 μM	2 μL	1.45 (±0.0094)	5.61 (±0.525)	5.83	−7.45	−13.3
3	PG, 4 mM	Rom, 100 μM	1 μL	1.29 (±0.086)	0.542 (±1.06)	5.37	−8.89	−14.3
Avg				1.49 (±0.047)	2.30 (±0.747)	6.10	−8.35	−14.47

a *n*, binding stoichiometry of the interaction of the two molecules; *K_D_*, dissociation constant; Δ*H*, enthalpy changes; Δ*G*, Gibbs free energy changes; Δ*S*, entropy changes.

### RomR shows no cross-resistance to various tested classical antibiotics.

Another interesting question was whether the massive excretion of lipids/fatty acids in RomR also causes cross-resistance to other antibiotics. For this reason, we compared the MICs for HG001 and its RomR mutant with those of 21 other antibiotics. As shown in Table 4, we observed no cross-resistance with any of the tested antibiotics. Therefore, Rom appears to have a unique ability to interact with PGs, resulting in the abrogation of its antimicrobial activity.

**TABLE 4:** MICs of various antibiotics for S. aureus HG001 and its RomR mutant

Antibacterial agent	MIC (μg/mL)	
	HG001	RomR
Rhodomyrtone	1	>128
Bacitracin	32	32
Benzalkonium chloride	2	2
Chloramphenicol	32	32
Daptomycin	1	1
Ethambutol	>32	>32
Gallidermin	2	2
Gentamicin	2	2
Gramicidin S	4	4
Hygromycin B	32	32
Kanamycin	8	8
Methicillin	4	4
Neomycin	0.5	0.5
Norfloxacin	1	1
Oxacillin	0.25	0.25
Penicillin G	0.03125	0.03125
Polymyxin B	256	256
Spectinomycin	>32	>32
Streptomycin	16	16
Sulfamethoxazole	32	32
Tunicamycin	>32	>32
Vancomycin	1	1

## DISCUSSION

The main goal of this work was to further decipher the mechanism of high Rom resistance in the RomR mutant. Originally, we thought that the point mutation in *farR* causing the amino acid change Cys116Arg would inactivate FarR* (12). But here we show that this is apparently not the case. FarR* still acts as a regulator, however, with altered activity. Transcriptome sequencing (RNA-seq) analysis showed that *farE* and the farR* gene are upregulated in RomR, thus resulting in a positive-feedback loop in which FarR* increases its own and FarE expression (12). In a closed system like a cell, such a hypercycle cannot continue endlessly. Therefore, the question arises as to the possible limiting factors. We assume that FarE, as a transmembrane protein, cannot be highly expressed without causing membrane jamming and cell damage. Therefore, we assume that FarE expression is tightly controlled to counteract the threat of cell damage by unlimited FarE expression. However, FarE appears to be advantageous for growth under certain conditions since in defined minimal medium, growth of the HG001 Δ*farE* and RomR Δ*farE* mutants was somewhat delayed (Fig. S5). Furthermore, FarR* upregulates not only *farE* and farR* but also several *agr*-controlled genes, which in addition could contribute to some of the effects observed.

There are two pieces of evidence that overexpression of FarE is responsible for the resistance to Rom: (i) upregulation of FarE and Rom resistance is correlated, and (ii) deletion of *farE* resensitizes the RomR mutant to Rom (Table 1). However, what we did not fully understand was the underlying mechanism: does FarE act as an efflux pump for Rom as described for the antimicrobial FAs (14), or is FarE rather an efflux pump of PGs in such large amounts that they can cause neutralization the antimicrobial activity of Rom?

To answer this question, we carried out comparative exolipidome analyses in S. aureus strains HG001, RomR, HG001Δ*farE*, and RomR Δ*farE* by analyzing the FAs, lipids, PG, Lys-PG, DG, MGDG, and DGDG both analytically and quantitatively. Since we know that S. aureus is able to take up and incorporate unsaturated FAs present, for example, on human skin (C16:1, C18:1, or C18:2) into PGs and the lipid moiety of lipoproteins (16), we cultivated the cells in defined minimal medium to exclude medium-derived FAs/lipids and their potential incorporation. We observed an enormous difference particularly in the release of PG and Lys-PG between RomR and its Δ*farE* mutant or HG001 (Fig. 2 and Table 2), with 10 times more excretion in the RomR strain than in the others. Therefore, we assume that FarE is primarily an efflux pump for PGs. We also observed an approximately 3- to 4-fold increase of FAs in RomR; however, the total amount of released FAs was >100 times lower than that of PGs. Our data suggest that FarE functions primarily as an exporter (efflux pump) of PGs.

There are other examples in the literature describing efflux pumps that excrete larger compounds. In Pseudomonas fluorescens, EmhABC excretes hydrophobic antibiotics, dyes, and polycyclic aromatic hydrocarbons, including phenanthrene (19). Two possible functions were discussed: that the efflux of FAs is a result of membrane damage or that the primary physiological role of the EmhABC efflux pump is the PG turnover. For Acinetobacter baumannii, a novel membrane protection system has been described, the AdeIJK efflux system, which modulates the lipid content of the membrane via direct efflux of lipids, probably contributing to membrane maintenance (20). The papers allude to the possibility that the efflux pumps might have PG as a substrate but did not explicitly state so.

While DG and MGDG were also present in the exolipidome, no difference was observed between RomR and RomR Δ*farE* or HG001. Similarly, only slightly larger amounts of DGDG were observed in the supernatant of RomR (Table 2). In S. aureus, DGDG serves as a membrane anchor molecule for lipoteichoic acid (LTA) (21, 22). The membranes of S. aureus contain 8 mol% of the free glycolipid, and the ratio of MGDG to DGDG may play an important role in determining bilayer stability, with only the latter forming a bilayer (23).

We could only detect saturated FAs in the exolipidome analysis, which is consistent with the absence of a fatty acid desaturase (24, 25). We could not discriminate between straight-chain saturated fatty acids (SCSFAs) and branched-chain fatty acids (BCFAs), with the latter playing a critical role in maintaining membrane fluidity (24, 26). Although S. aureus encodes cardiolipin synthases 1 and 2, we did not detect cardiolipin, most likely because we harvested the supernatant at the end of the exponential growth phase and it has been reported that cardiolipin is produced mainly in the stationary phase (27).

Although we had ample evidence that Rom can be neutralized by PGs, we attempted to show a direct interaction. This experiment was complicated by the fact that both compounds were insoluble in water. As a model PG, we chose PG(32:0). Using the methods described, it was finally possible to dissolve both compounds in 10% DMSO/PBS (pH 7.2). Using SLS/DLS analysis, we could demonstrate that in freshly prepared solutions, both components formed vesicles with mean sizes at a 90° angle of 90.8 nm for PG(32:0) and of 120 nm for Rom. When the two components were mixed, the mean size of 100 nm at the beginning increased with time to 200 nm (Fig. 5C), which is a first hint that Rom and PG(32:0) vesicles interact with each other. So far it is unknown in what form the secreted PG(32:0) is present in the culture supernatant. However, since the environment is predominantly aqueous, PG(32:0) should also form vesicles with time. The vesicle formation is most likely concentration dependent, similar to the case with the critical micelle concentration (CMC) for surfactants, detergents, and PGs. For example, the CMC for various PGs was in the range of 0.6 to 3.7 μM (28). With a concentration of 1.0 mM PG(32:0), this is well above the CMC. In the supernatant of the RomR mutant, we obtained 100 to 200 μM for each of PG(30:0), PG(31:0), and PG(32:0), excluding the less abundant PG(29:0), PG(33:0), and PG(34:0). All PGs together in the culture supernatant of the cells at the end of the exponential growth phase amounted to about 600 μM (Fig. S6). This means that with a concentration of 1 mM PG(32:0), our results were well within a realistic range.

10.1128/mbio.03833-21.6 FIG S6 Lipidomic analysis in supernatants only for PGs in HG001, RomR, HG001Δ*farE*, and RomR Δ*farE* in the absence and presence of Rom. This illustration is similar to the one in Fig. 2 in the main text. The only difference is that here, we show the molarity (and not nanograms/OD) of PGs in the supernatant. The PGs ranged from C28:0 to C36:0. It is observed that the presence of a sublethal concentration of Rom did not alter much the PG release. Download FIG S6, PDF file, 0.1 MB. Copyright © 2022 Huang et al. 2022 Huang et al. https://creativecommons.org/licenses/by/4.0/ This content is distributed under the terms of the Creative Commons Attribution 4.0 International license.

In order to directly analyze the interaction of Rom with PG(32:0), we used isothermal titration calorimetry (ITC). ITC can measure the association constant (*K_a_*), reaction stoichiometry (*n*), the heat capacity (ΔCp) of the reaction, binding free energy (Δ*G*), entropy (Δ*S*), and enthalpy (Δ*H*). We obtained similar heat fluxes in all three experiments, each with slightly different conditions (Fig. 6). The results indicate that there is a rapid (millisecond) interaction of Rom with PG(32:0) which is entropically driven and hence hydrophobic and of low specificity in nature. The ratio of Rom binding to PG(32:0) was 1:1.49.

Another question is how to explain the high Rom resistance (MIC > 128 μg/mL) in the RomR mutant (Table 1). In the MIC analysis, a high concentration of Rom was added right at the beginning of the diluted cell culture; thus, there was insufficient time for the excretion of PG in the supernatant to reach a concentration high enough to neutralize Rom. Nevertheless, there was hardly a lag phase when Rom was added to the RomR mutant (Fig. S1). This indicates that the Rom resistance acted in the RomR mutant from the beginning. There are two possible explanations for this observation: (i) FarE acts as an efflux pump not only for PG but also for Rom, and (ii) in the RomR mutant, a high concentration of PG is already stored near the membrane and also in the cell wall, providing high resistance from the beginning, and therefore, we see almost no lag phase in the RomR mutant. In fact, the concentration of PG in the pellet wash was almost as high as in the supernatant (Fig. 2), indicating that a high proportion of PG is accumulated in the cell envelope. Therefore, we hypothesize that the second is the most plausible explanation, since it is unlikely that FarE also serves as an efflux pump for Rom; the structures of PG and Rom are too different.

We tested several clinically applied antibiotics and could not detect any cross-resistance (Table 3). There are only a few antimicrobial compounds reported that interact with PGs. One is the positively charged antibiotic gentamicin, which interacts with cell membranes, especially PGs (29–31). This interaction induced membrane permeabilization and depolarization, the same activity as we also observed with Rom (10). The other antibiotic is daptomycin, which is inactivated by the released membrane PGs (32). It was a bit surprising that the MIC and the minimum bactericidal concentration (MBC) were not increased in the RomR mutant, which accumulated a high concentration of PGs (≈600 μM) in the culture supernatant (Fig. S6). The new insight that Rom binds to P-lipids leads us to assume that the cytoplasmic membrane is the actual target of Rom. This is in agreement with our previous findings that Rom causes a collapse of the membrane potential within seconds and induces local membrane damage. Therefore, the actual mechanism of resistance in the RomR mutant is overproduction of the target molecule. Resistance mechanisms based on overexpression of the target molecule are rare but have been described for some antibiotics. For example, one of the several mechanisms of trimethoprim resistance is based on overexpression of the target enzyme dihydrofolate reductase (33). The second main cause of resistance to isoniazid (INH) is the overexpression of enoyl-acyl-carrier protein reductase InhA (34, 35). Overexpression of the d-alanine racemase gene confers resistance to d-cycloserine in Mycobacterium smegmatis (36).

So far, plants are not known to produce small antimicrobial compounds that bind PGs. To the best of our knowledge, Rom is a novelty. From the immunology point of view, antibodies that are directed against PGs and PG-binding proteins play a role in certain human diseases. Too much of anti-PG antibodies, such as in lupus anticoagulant, anti-cardiolipin antibodies, and anti-β2-glycoprotein 1 antibodies, can cause the so-called antiphospholipid syndrome (APS), an autoimmune disease occurring mostly in young women (37). It would be worthwhile to examine the impact of Rom on APS in more detail.

### Conclusion.

**FIG 7:**
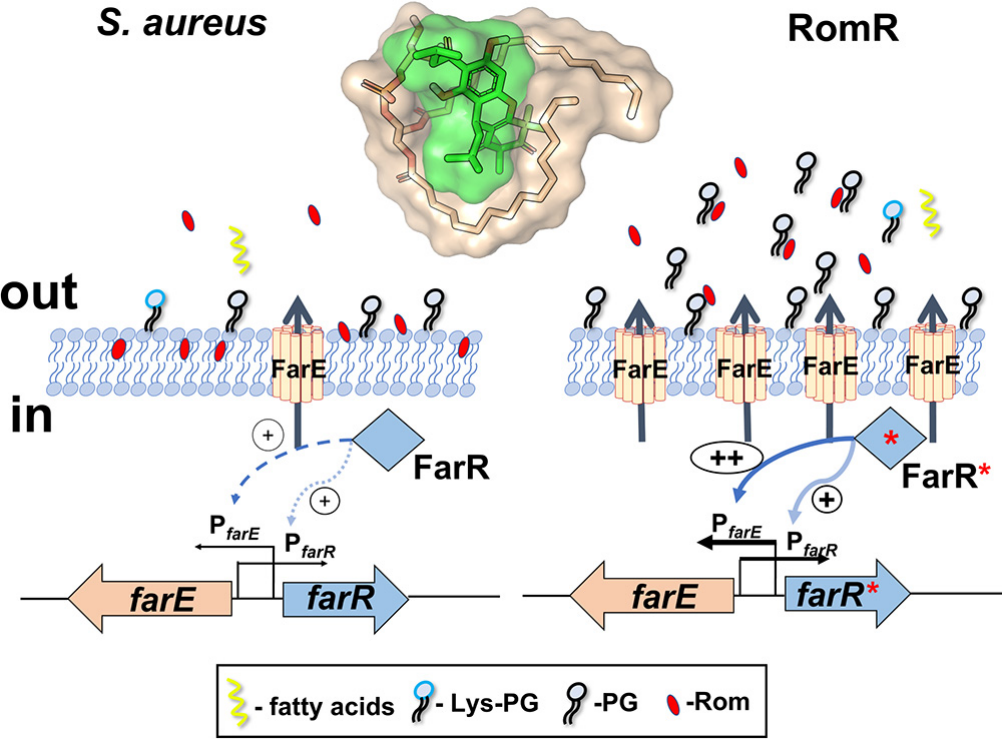
Mechanism of Rom resistance (graphical abstract). The regulation of *farE* and *farR* as well as the secretion of PGs in the parental strain S. aureus HG001 in comparison with its RomR mutant is illustrated. In HG001, FarR mildly activates *farE* and *farR* expression. The membrane-localized FarE secretes small amounts of PGs, which are too small to cause resistance to Rom. In the RomR mutant, FarR* becomes a potent activator of *farE* and *farR*. The resulting overexpression of FarE, acting as a PG efflux pump, leads to increased accumulation of PGs in the supernatant and cell envelope. The PG concentration is now high enough to efficiently scavenge Rom, thereby abrogating its antimicrobial activity. Rom binds to PGs at a ratio of 1:1.4. We hypothesize that the resistance results from interaction of Rom with PGs, causing neutralization of Rom’s antimicrobial activity. The inserted structure of PG and Rom shows a possible interaction of both compounds.

Considering the results of our lipidomic analysis showing that the RomR mutant excretes much larger amounts of PGs than its parent strain and that Rom indeed binds to PG, we provide strong evidence that Rom resistance in the RomR mutant is due to the neutralization of Rom’s activity by binding to PGs. Our hypothesis for the *farE-farR* function is the following. In the wild type, *farE* expression is low and FarR acts only as a mild activator. In the RomR mutant, the point mutation in farR* causes FarR* to become a strong activator of *farE* and its own (farR*) gene expression. Overexpression of FarE, which acts most likely as a PG efflux pump, causes massive accumulation of PG in the supernatant and the cell envelope area. PG scavenges Rom, thereby abrogating its antimicrobial activity (Fig. 7).

## MATERIALS AND METHODS

### Bacterial strains and growth conditions.

Bacterial strains and plasmids used in this study are listed in Table S1. For cloning procedures, S. aureus and Escherichia coli strains were grown in basic medium (BM) containing 1% (wt/vol) soy peptone, 0.5% yeast extract, 0.5% NaCl, 0.1% K_2_HPO_4_, and 0.1% glucose at pH 7.2. Bacteria were cultivated aerobically (200 rpm) at 37°C. For lipidomic analysis, we used a defined minimal medium as described by Rudin et al. (38). To investigate whether Rom has an impact on excretion of lipids/fatty acids in S. aureus, we supplemented the medium with sublethal concentrations (0.3 μg/mL) of Rom.

10.1128/mbio.03833-21.7 TABLE S1 Bacterial strains and plasmids used in this study. Download Table S1, DOCX file, 0.02 MB. Copyright © 2022 Huang et al. 2022 Huang et al. https://creativecommons.org/licenses/by/4.0/ This content is distributed under the terms of the Creative Commons Attribution 4.0 International license.

### Construction of deletion mutants.

The deletion mutants were constructed as markerless deletions as previously described (12). Recombinant knockout plasmids were constructed using the temperature-sensitive plasmid pBASE6, which is a derivative of pBT2 (39, 40). Briefly, ∼1,000-bp upstream and ∼1,000-bp downstream fragments of SAOUHSC_02866 (*farE*) and SAOUHSC_01359 (*mprF*) were amplified from the genomic DNA of S. aureus HG001 and RomR strains, respectively. Then, the fragments were assembled with the EcoRI-linearized pBASE6 using Hi-Fi DNA assembly master mix (New England BioLabs). The ligation mixtures were transformed into E. coli DC10B chemically competent cells. The correct plasmids were confirmed by PCR and sequencing before being transformed via electroporation into HG001 and RomR, respectively. Mutagenesis was performed as previously described (41, 42). The mutants were named S. aureus HG001 Δ*farE*, RomR Δ*farE*, and RomR Δ*mprF*. All oligonucleotides used in this study are listed in Table S2.

10.1128/mbio.03833-21.8 TABLE S2 Oligonucleotides used in this study. Download Table S2, DOCX file, 0.01 MB. Copyright © 2022 Huang et al. 2022 Huang et al. https://creativecommons.org/licenses/by/4.0/ This content is distributed under the terms of the Creative Commons Attribution 4.0 International license.

### Determination of MIC.

MIC values of Rom were determined in 96-well microtiter plates using BM as previously described (43). Briefly, 50 μL of Rom was serially diluted from 128 μg/mL to 0.25 μg/mL. Then, 50 μL of bacterial culture (10^6^ CFU/mL) was added to each well. An inoculated broth without Rom was regarded as a positive control, and the well without bacteria was used as a negative control. The 96-well microtiter plates were incubated at 37°C for 24 h. The MIC values were determined as the lowest concentration that completely inhibited the visible growth of bacteria. The experiments were repeated three times.

### Growth studies of staphylococcal strains in defined minimal medium.

For lipidomic analysis, bacteria were cultivated with shaking at 37°C in defined medium using a 48-well Varioskan Lux microplate reader (Thermo Scientific). Bacteria were precultured in defined medium inoculated into 500 μL of fresh defined medium at a starting OD_578_ of 0.1, and growth of HG001, HG001 Δ*farE*, RomR, and RomR Δ*farE* was monitored for 48 h (Fig. S3).

### Impact of supernatants of HG001 and RomR on Rom activity.

HG001 was inoculated into BM and incubated at 37°C with shaking overnight, and the impact of Rom (2 μg/mL) and culture supernatant (S) on growth was monitored. Culture supernatants from the HG001 and RomR strains were harvested at the end of the exponential growth phase, sterile filtered (0.2-μm filter), and concentrated 10 times with a speed vacuum concentrator. The supernatant was applied at 1:10 (vol/vol) (Fig. 1B).

### Preparation of the samples for lipidomic analysis.

The overnight bacterial cultures (HG001, HG001 Δ*farE*, RomR, and RomR Δ*farE*) were used to inoculate 15 mL of defined minimal medium in 100-mL flasks (OD_578_ = 0.1) and incubated at 37°C with shaking until the end of the exponential phase was reached. Cells were then centrifuged at 8,000 rpm and 4°C for 10 min, and both the supernatants and pellets were collected. The supernatants were filter sterilized and lyophilized. All samples were prepared in five biological replicates and used for subsequent preparation for lipidomic analyses. We also investigated whether sublethal concentrations of Rom can induce lipid/FA release. In this case, the cultures were grown in the presence of 0.3 μg/mL of Rom.

### Materials used in the isolation and analyses of lipids and fatty acids (internal standard [IS]).

SPLASH LIPIDOMIX mass spectrometry standard, 18:2 cardiolipin-d5 (18:2 CL-d5), 18:0 phosphatidylglycerol-d70 (18:0 PG-d70), and 18:1 lysyl-phosphatidylglycerol (18:1 Lys-PG) were purchased from Avanti Polar Lipids (Alabaster, AL). Arachidonic acid-d11 (AA-d11) and C18-ceramide-d7 (d18:1-d7/18:0) were obtained from Cayman Chemicals (Ann Arbor, MI). Isopropanol (IPA), acetonitrile (ACN), and methanol (MeOH) at ultra-LC-MS grade were from Carl Roth (Karlsruhe, Germany). Ammonium formate, formic acid, and IPA at high-performance liquid chromatography (HPLC) grade were purchased from Merck (Darmstadt, Germany). Purified water was produced by Elga Purelab Ultra (Celle, Germany).

### Lipid extractions from bacterial samples.

Lipid extraction from bacterial supernatant was performed by a biphasic extraction method following the protocol of Matyash et al. (44). First, the IS mixture was prepared by mixing 75 mL of ice-cold MeOH with 250 μL of LipidoMIX solution, 5 μL of AA-d11 stock solution (1 mg/mL), 50 μL of 18:2 CL-d5 stock solution (1 mg/mL), 50 μL of d18:1-d7/18:0 stock solution (0.25 mg/mL), 50 μL of 18:0 PG-d70 (1 mg/mL), and 50 μL of Lys-PG(18:1) (1 mg/mL) stock solutions. The IS mixture was then fully vortexed, and 1.5 mL of ice-cold methanol containing IS was added to a 50-mL Falcon tube with freeze-dried bacterial supernatant for each sample. The samples were vortexed for 10 s. Afterwards, 5 mL of ice-cold methyl tert-butyl ether (MTBE) was added. Samples were incubated on ice for 1 h, followed by addition of 1.25 mL of H_2_O to account for a final ratio of MTBE-MeOH-H_2_O of 10:3:2.5 (vol/vol/vol) and incubation at room temperature for another 10 min to induce phase separation. The upper layer was transferred to a new Falcon tube, and the water phase was reextracted by adding 2 mL of the upper phase from a solution of MTBE-MeOH-H_2_O (10:3:2.5 [vol/vol/vol]). The upper layer from reextraction was then combined with the phase from first extraction and dried with a Genevac EZ-2 evaporator (SP, Ipswich, UK) with nitrogen protection. Extraction residues after evaporation were reconstituted in 100 μL of MeOH, and after vortexing (10 s), sonication (2 min), and centrifugation (3,500 × *g*, 10 min), the methanol solutions were transferred to autosampler vials.

For the preparation of bacterial pellet wash, lipid extraction was performed using a monophasic extraction method following the IPA/H_2_O protocol (45). An IS mixture was prepared by mixing 450 mL of ice-cold IPA and 50 mL of H_2_O with 500 μL of LipidoMIX solution, 10 μL of AA-d11 stock solution (1 mg/mL), 100 μL of CL-d5(18:2) stock solution (1 mg/mL), 100 μL of d18:1-d7/18:0 stock solution (0.25 mg/mL), 100 μL of 18:0 PG-d70 (1 mg/mL), and 100 μL of Lys-PG(18:1) (1 mg/mL) stock solutions. Dry bacterial pellets were suspended in 5 mL of IPA/H_2_O (9:1 [vol/vol]) with IS, vortexed for 10 s, and sonicated for 2 min. Then the samples were incubated on ice for 1 h with 2 min of sonication every 12 min during the incubation, which means a total of 5 cycles of sonication (2 min). The samples were centrifuged (3,500 × *g*, 10 min), pellets were kept, and supernatant (lipid extract) was transferred to fresh Falcon tubes and dried with the Genevac EZ-2 evaporator (SP, Ipswich, UK) with nitrogen protection. Afterwards, the extracts were reconstituted in 100 μL of MeOH, vortexed (10 s), sonicated (2 min), centrifuged (3,500 × *g*, 10 min), and transferred to autosampler vials.

Lastly, for the extraction from bacterial pellets, 5 mL of IPA/H_2_O (9:1 [vol/vol]) with IS was added to the pellets, which were kept after washing from the previous step. Samples were vortexed and sonicated. After adding beads (1-mm diameter) and enzyme (50 μL of lysostaphin at 0.3 mg/mL) to each sample, the pellets were disrupted in a Fastprep-24 (MP Biomedicals) (3 cycles of 30 s each at a speed 6.5 m/s). Samples were then centrifuged (3,500 × *g*, 10 min), and supernatant was collected for further drying with a Genevac EZ-2 evaporator (Ipswich, UK) with nitrogen protection. The dried extracts were then reconstituted, vortexed, sonicated, and transferred into vials as described above. A pooled quality control (QC) sample was prepared by mixing 15-μL aliquots of each reconstituted sample from three extraction process.

### UHPLC-ESI-QTOF-MS/MS method.

The analysis of samples was performed with an Agilent 1290 Infinity ultrahigh-performance liquid chromatography (UHPLC) system (Agilent, Waldbronn, Germany) equipped with a binary pump and a PAL-HTX xt DLW autosampler (CTC Analytics AG, Switzerland) and coupled to a SCIEX TripleTOF 5600+ quadrupole time of flight (QTOF) mass spectrometer with a DuoSpray source (SCIEX, Ontario, Canada). The chromatographic separation was performed on an Acquity UPLC CSH C_18_ column (100 mm by 2.1 mm; 1.7-μm particles; Waters Corporation, Milford, MA) with precolumn (5 mm by 2.1 mm; 1.7-μm particles). The column temperature was 65°C, with a flow rate 0.6 mL/min. Mobile phase A was composed of H_2_O/ACN (2:3 [vol/vol]) containing 10 mM ammonium formate and 0.1% (vol/vol) formic acid, while mobile phase B was IPA/ACN/H_2_O (90:9:1 [vol/vol/vol]) containing 10 mM ammonium formate and 0.1% (vol/vol) formic acid. A gradient elution started from 15% mobile phase B to 30% mobile phase B in 2 min, followed by an increase of mobile phase B to 48% in 0.5 min. Then mobile phase B was further increased to 82% at 11 min and quickly reached 99% in the next 0.5 min, followed by holding this percentage for another 0.5 min. Afterwards, the percentage of mobile phase B was taken back to starting conditions (15% mobile phase B) in 0.1 min to reequilibrate the column for the next injection (2.9 min).

LC-ESI-MS/MS experiments were operated in both positive and negative modes with injection volumes of 3 μL for positive and 5 μL for negative mode. An MS full-scan experiment with mass range *m/z* of 50 to 1,250 was selected, while different SWATH windows were acquired for MS/MS experiments (Table S3). The ion source temperature was set to 350°C with curtain gas (CUR), nebulizer gas (GS1), and heater gas (GS2) pressures 35 lb/in^2^, 60 lb/in^2^, and 60 lb/in^2^, respectively, for both modes. The ion spray voltage was set to 5,500 V in the positive mode and −4,500 V in negative mode. The declustering potential (DP) was adjusted to 80 V and −80 V for positive and negative polarity modes, respectively. The cycle time was always 720 ms. The collision energy (CE) and collision energy spread (CES) for each experiment are shown in Table S3. The sequence was started with three injections of IS mixture as a system suitability test. The whole sequence was controlled by injection of QC samples after every five samples.

10.1128/mbio.03833-21.9 TABLE S3 MS/MS experiment of SWATH windows with *m/z* range, accumulation time (Acc. time), and collision energy. Download Table S3, DOCX file, 0.02 MB. Copyright © 2022 Huang et al. 2022 Huang et al. https://creativecommons.org/licenses/by/4.0/ This content is distributed under the terms of the Creative Commons Attribution 4.0 International license.

### Effects of FAs, lipids, and PGs on Rom activity.

The effects of FAs (pentadecanoic acid, palmitic acid, and stearic acid) and PGs [PG(18:0) and Lys- PG(18:1)] on Rom activity were determined in a 48-well microplate reader in BM at 37°C with shaking overnight. The OD_578_ was determined every 2 h for 24 h. All experiments were conducted in three independent biological replicates.

### Solubilization of Rom and PG(32:0).

Since PG(32:0) was insoluble in all organic solvents tested, an organic solvent mixture (chloroform- methanol-water at 65:35:8 [vol/vol/vol]) was used to dissolve it. However, this solvent mixture was not suitable to be used for ITC because of the volatilization of chloroform; thus, PG(32:0) was prepared as vesicles formed in 10% DMSO/PBS and used for ITC and DLS. Rom, on the other hand, was soluble in all organic solvents tested but not in water; thus, it was first dissolved in 100% DMSO and subsequently diluted in 10% DMSO/PBS. Thus, both compounds were dissolved in the same solvent, which is a prerequisite for ITC studies.

### Preparation of PG vesicles and determination of size distribution by DLS.

PG(32:0), 1,2-dipalmitoyl-*sn*-glycero-3-phospho-(1′-rac-glycerol) (sodium salt), was purchased from Avanti Polar Lipids Inc. PG powder was dissolved in organic solvent (chloroform-methanol-water at 65:35:8 [vol/vol/vol]) in a glass tube. The lipid was dried completely by evaporator for 2 h and exicator overnight. The dried lipid film was weighed, dissolved in buffer (10% DMSO/PBS), adjusted to a concentration of 5 mM, and treated in an ultrasonic bath for 2 h. The lipid dispersion was transferred to an Eppendorf tube and subjected to ultrasonication (Titan tip) four times, each for 2 min. Subsequently, the size distribution of the vesicles was determined by DLS.

### ITC.

ITC experiments were performed using a MicroCal PEAQ-ITC calorimeter (Malvern). PG(32:0) vesicles and Rom were dissolved in 10% DMSO/PBS (1:9 [vol/vol]) and degassed via vacuum before titration. An initial volume of 0.4 μL followed by 18 injections of 2 μL of PG(32:0) vesicles was injected into the cell with Rom solution. Injection of PG(32:0) vesicles into 10% DMSO was used as a control to correct the heat of dilution. The measurements were carried out at 37°C with stirring at 750 rpm with 150-s intervals. The final data were analyzed via the “one-site-binding” model to determine the binding affinity (*K_D_*), enthalpy (Δ*H*) and entropy (Δ*S*) of binding and stoichiometry (*n*).

### Static and dynamics light scattering (SLS/DLS).

The SLS/DLS experiments were performed using an ALVCGS3 setup with a wavelength of 632.8 nm. The CONTIN analysis was performed using the light scattering software provided by ALV. Toluene and water were used as the standard and solvent for all measurements. The hydrodynamic radius obtained at 90° was used to describe the size of PG(32:0) vesicles and Rom clusters.

### Data visualization and analysis.

The growth curves and bar charts were visualized using GraphPad Prism 6.0 software. Mann-Whitney U test was applied to compare significant differences between HG001 and RomR. R was employed for performing the Mann-Whitney U test.

### Data availability.

The main data supporting the findings of this work are available within the article and the supplemental material files or from the corresponding author upon request.
