# Supplementary material for: Molecular Basis of Rhodomyrtone Resistance in Staphylococcus aureus
Source: mBio. 2022 Feb 15;13(1):e03833-21. doi: 10.1128/mbio.03833-21 (PMC8844917; doi:10.1128/mbio.03833-21)
Supplement: FIG S1 [file mbio.03833-21-sf001.pdf]

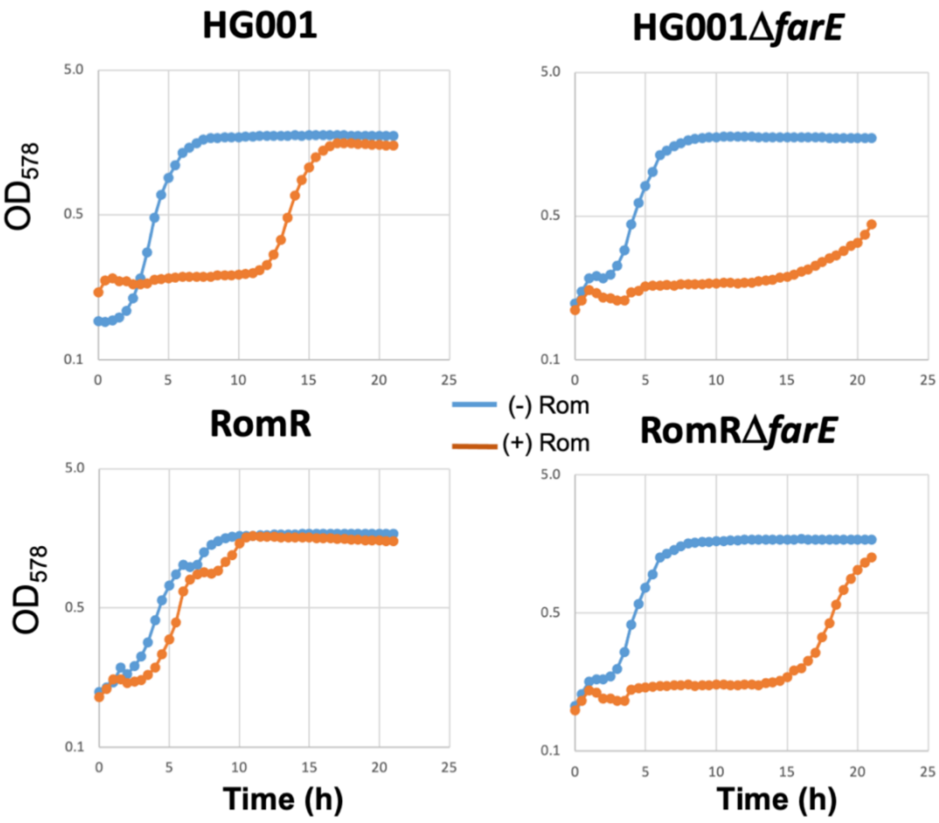

Figure S1. Impact of Rom on the growth of HG001 and RomR and their respective Δ*farE* mutants. The growth of the *S. aureus* clones in BM supplemented with Rom (8 μg/ml) was followed for 24 h. Growth of HG001 was inhibited for about 12 h but resumed thereafter; growth of HG001Δ*farE* was inhibited for the whole period. Growth of RomR mutant was unaffected in the presence of Rom (completely resistant to Rom), while the growth of RomRΔ*farE* was inhibited for about 15 h, and resumed thereafter. The result indicate that FarE is crucial for Rom resistance. The graph shown here is a representative of one of the triplicates growth studies performed.
