# Supplementary material for: Molecular Basis of Rhodomyrtone Resistance in Staphylococcus aureus
Source: mBio. 2022 Feb 15;13(1):e03833-21. doi: 10.1128/mbio.03833-21 (PMC8844917; doi:10.1128/mbio.03833-21)
Supplement: FIG S2 [file mbio.03833-21-sf002.pdf]

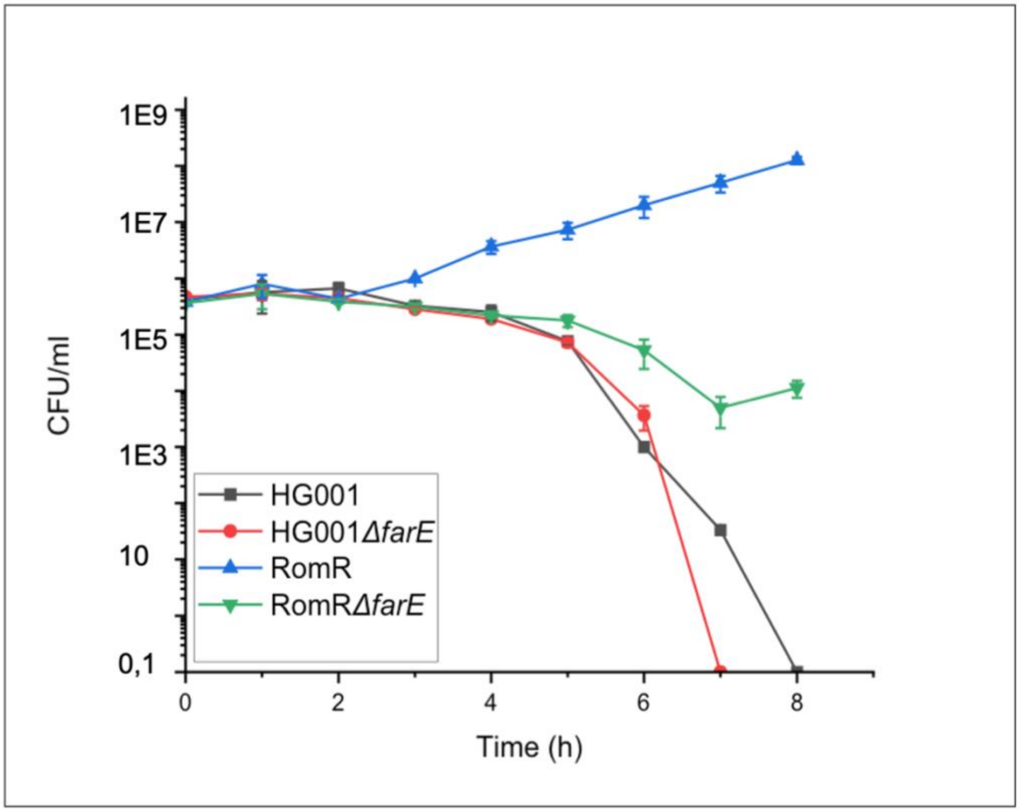


**Figure S2. Impact of Rom on the killing of HG001 and RomR and their respective Δ*farE* mutants.** The killing of the *S. aureus* clones in BM supplemented with Rom (8 μg/ml) was followed for 8 h. Cells were inoculated to a cell concentration of about 6 x $10^5$ cfu/ml. HG001 and HG001Δ*farE* were almost completely killed after 7-8 h, while growth of the RomR mutant continued. With RomRΔ*farE* mutant, killing was also observed, but it was markedly delayed compared to HG001 and HG001Δ*farE*. Each bar represents the mean ± SD from three independent biological replicates.
