# Supplementary material for: Molecular Basis of Rhodomyrtone Resistance in Staphylococcus aureus
Source: mBio. 2022 Feb 15;13(1):e03833-21. doi: 10.1128/mbio.03833-21 (PMC8844917; doi:10.1128/mbio.03833-21)
Supplement: FIG S3 [file mbio.03833-21-sf003.pdf]

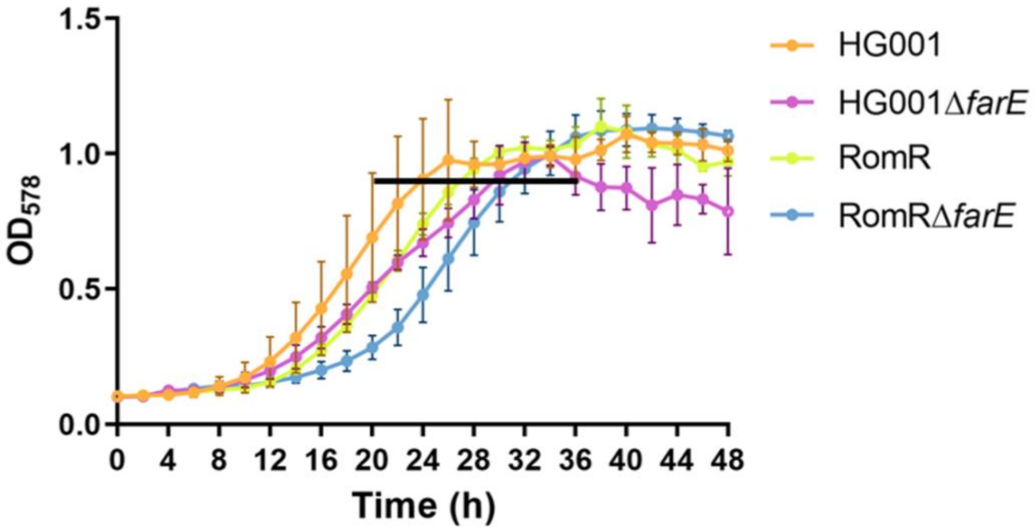

Figure S3. Comparative growth of HG001 and its mutants in defined minimal medium (AAM) using a microplate reader. Each point in the graph is the mean ± SD of three independent biological replicates; bar, indicates end-exponential phase ($OD_{578} \approx 0.9$) at which the cells were harvested for lipidomic analysis.
