# Supplementary material for: Molecular Basis of Rhodomyrtone Resistance in Staphylococcus aureus
Source: mBio. 2022 Feb 15;13(1):e03833-21. doi: 10.1128/mbio.03833-21 (PMC8844917; doi:10.1128/mbio.03833-21)
Supplement: FIG S4 [file mbio.03833-21-sf004.pdf]

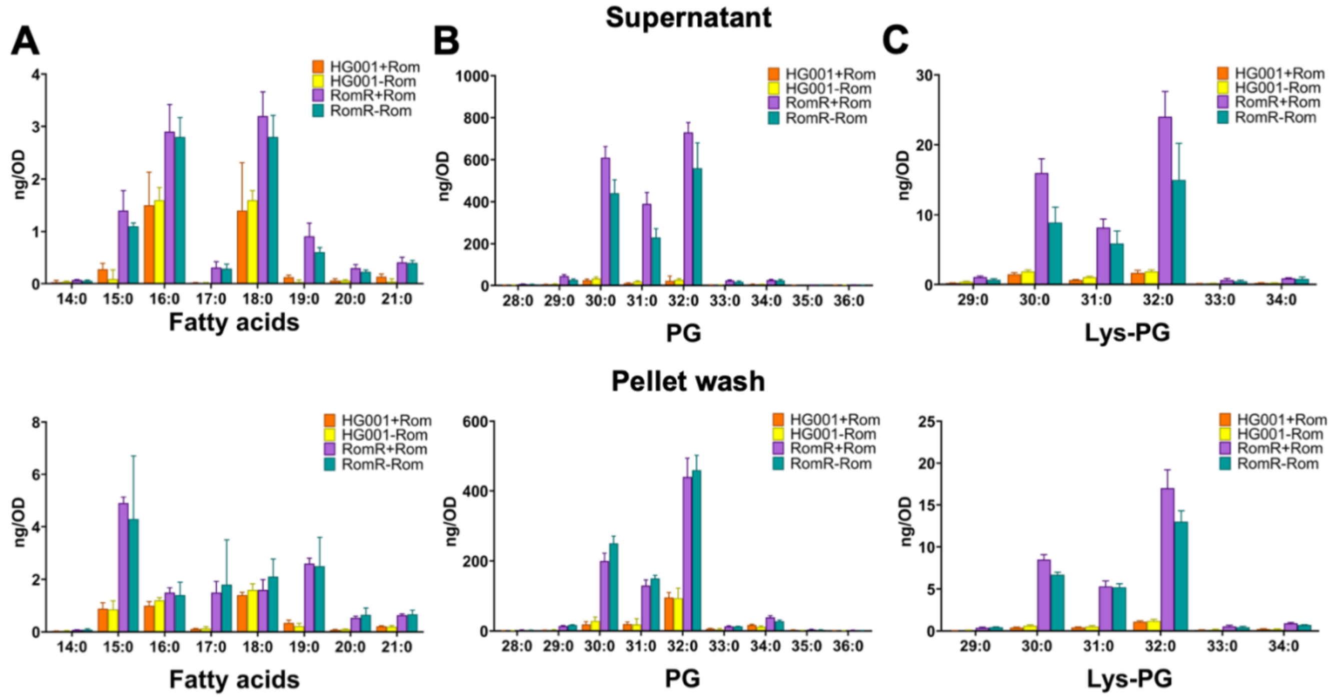

Figure S4. Effect of sub-inhibitory concentrations of Rom on the release of fatty acids and lipids by the *S. aureus* HG001 and RomR strains in the supernatant (above) and pellet wash (below). The bar charts show the amount of (**A**) fatty acids ranging from C14:0 to C21:0 (**B**) phosphatidylglycerol (PG) ranging from C28:0 to C36:0 and (**C**) lysyl phosphatidylglycerol (Lys-PG) ranging from C29:0 to C:34 detected in both the strains in the presence of sub-inhibitory concentration of Rom at 0.3 µg/ml. All absolute amounts of FAs and lipids were reported in ng/OD_{578} of the bacterial cultures grown until end exponential phase and adjusted to the corresponding OD_{578} = 1.0. The supernatant and pellet were separated by centrifugation for lipidomic analyses. The pellet wash was obtained by treating the pellets with 90% isopropanol to remove the fatty acids and lipids from the surface of cells. Each bar shown in the chart represents the mean ± SD from five independent biological replicates.
