# Supplementary material for: Molecular Basis of Rhodomyrtone Resistance in Staphylococcus aureus
Source: mBio. 2022 Feb 15;13(1):e03833-21. doi: 10.1128/mbio.03833-21 (PMC8844917; doi:10.1128/mbio.03833-21)
Supplement: FIG S5 [file mbio.03833-21-sf005.pdf]

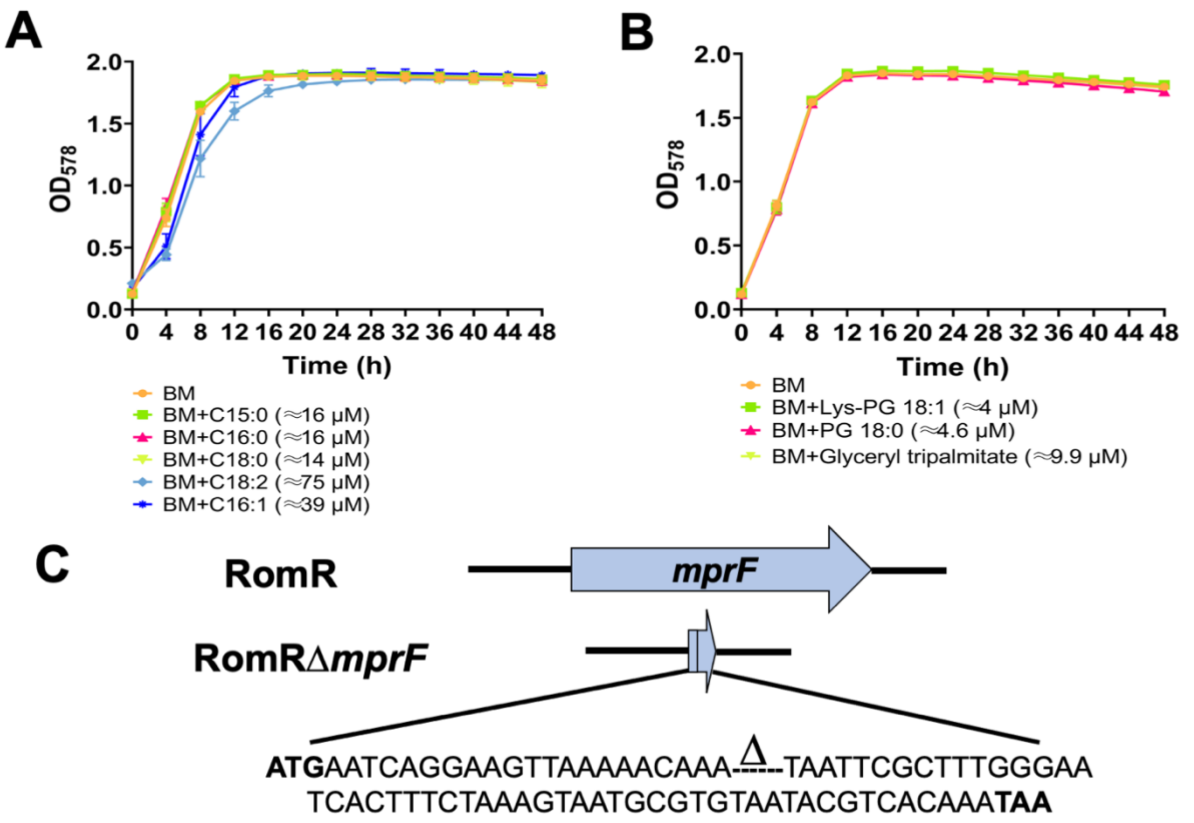


**Figure S5. Impact of exogenous supplementation of FAs / lipids on the growth of HG001 and construction of RomRΔ*mprF*.** The growth of *S. aureus* HG001 in BM with supplementations of (**A**) fatty acids at 4 µg/ml C15:0 (≈16 µM), 4 µg/ml C18:0 (≈14 µM), 4 µg/ml C16:0 (≈16 µM), 10 µg/ml C16:1 (≈39 µM) 21 µg/ml C18:2 (≈75 µM); (**B**) lipids at 4 µg/ml PG 18:0 (≈4.6 µM), 4 µg/ml Lys-PG 18:1 (≈4 µM). Well containing BM only and bacteria was regarded as positive control. Growth of the bacteria was measured at $OD_{578}$ every 4 h for 48 h using a microplate reader Varioskan Lux (Thermo Scientific) in a 48 wells plate. Each point in the graph is the mean ± SD from three independent biological replicates. (**C**) Illustration of RomRΔ*mprF* deletion mutant.
