# Supplementary material for: Molecular Basis of Rhodomyrtone Resistance in Staphylococcus aureus
Source: mBio. 2022 Feb 15;13(1):e03833-21. doi: 10.1128/mbio.03833-21 (PMC8844917; doi:10.1128/mbio.03833-21)
Supplement: FIG S6 [file mbio.03833-21-sf006.pdf]

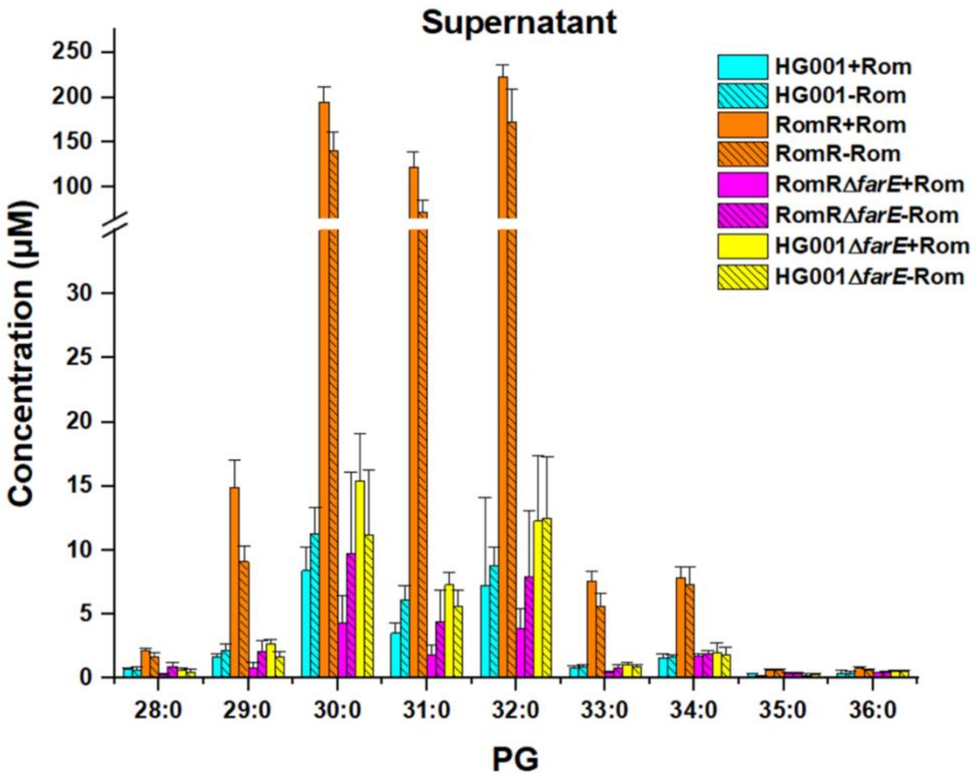

Figure S6. Lipidomic analysis in supernatants only for PGs in HG001, RomR, HG001Δ*farE* and RomRΔ*farE* in the absence and presence of Rom. This illustration is similar to the one in Fig. 2. The only difference is that here, we show the molarity (and not ng/OD) of PGs in the supernatant. The PGs ranged from C28:0 to C36:0. It is observed that the presence of sublethal concentration of Rom did not alter much the PG release.
