# Supplementary material for: Molecular Basis of Rhodomyrtone Resistance in Staphylococcus aureus
Source: mBio. 2022 Feb 15;13(1):e03833-21. doi: 10.1128/mbio.03833-21 (PMC8844917; doi:10.1128/mbio.03833-21)
Supplement: TABLE S1 [file mbio.03833-21-st001.docx]

**Supplementary Tables**

**Table S1: Bacterial strains and plasmids used in this study**

| **Strains** | **Description** | **Source** |
| --- | --- | --- |
| *S. aureus* HG001 | Derivative of *S. aureus* NCTC8325 with repaired *rsbU* | (1) |
| RomR | Derivative of HG001 which is Rom-resistant | (2) |
| HG001∆*farE* | Deletion of *farE* in HG001 | This study |
| RomR∆*farE* | Deletion of *farE* in RomR | This study |
| RomRΔ*mprF* | Deletion of *mprF* in RomR | This study |
| *E. coli* DC10B | Common laboratory strain for cloning | (3) |
| **Plasmids** |  |  |
| pBASE6 | Temperature-sensitive plasmid used for knockout | (4) |
| pBASE6_D_*farE* | Recombinant plasmid that is used for deletion of *farE* in HG001 | This study |
| pBASE6_D*_*farE* | Recombinant plasmid that is used for deletion of *farE* in Rom^R^ | This study |
| pBASE6_D_*mprF* | Recombinant plasmid that is used for deletion of *mprF* in Rom^R^ | This study |

**Supplementary reference for Table S1**

1. Herbert S, Ziebandt AK, Ohlsen K, Schafer T, Hecker M, Albrecht D, Novick R, Götz F. 2010. Repair of global regulators in *Staphylococcus aureus* 8325 and comparative analysis with other clinical isolates. Infect Immun 78:2877-89.

2. Nguyen MT, Saising J, Tribelli PM, Nega M, Diene SM, Francois P, Schrenzel J, Sproer C, Bunk B, Ebner P, Hertlein T, Kumari N, Härtner T, Wistuba D, Voravuthikunchai SP, Mader U, Ohlsen K, Götz F. 2019. Inactivation of *farR* Causes High Rhodomyrtone Resistance and Increased Pathogenicity in *Staphylococcus aureus*. Front Microbiol 10:1157.

3. Monk IR, Foster TJ. 2012. Genetic manipulation of Staphylococci–Breaking through the barrier. Frontiers in cellular and infection microbiology 2:49.

4. Geiger T, Francois P, Liebeke M, Fraunholz M, Goerke C, Krismer B, Schrenzel J, Lalk M, Wolz C. 2012. The stringent response of *Staphylococcus aureus* and its impact on survival after phagocytosis through the induction of intracellular PSMs expression. PLoS Pathog 8:e1003016.
