# Supplementary material for: Molecular Basis of Rhodomyrtone Resistance in Staphylococcus aureus
Source: mBio. 2022 Feb 15;13(1):e03833-21. doi: 10.1128/mbio.03833-21 (PMC8844917; doi:10.1128/mbio.03833-21)
Supplement: TABLE S2 [file mbio.03833-21-st002.docx]

**Table S2:** Oligonucleotides used in this study

| **Primer name** | **Sequence (5’→3’)** |
| --- | --- |
| Construction of HG001Δ*farE* and RomRΔ*farE* | |
| up_*farE*_F | CACTCATCGCAGTGCAGCGGATAGCAACCATATTTTTAGAAAAG |
| up_*farE*_R | TTTATGATTTTGTCCCCATCTTATATAAAAATTTTG |
| down_*farE*_F | GATGGGGACAAAATCATAAAATAACATGTACATGC |
| down_*farE*_R | GCCCGGGTACCGAGCTCCGGATGATTGCGTATACGCTC |
| Construction of RomRΔ*mprF* | |
| up_*mprF*_F | CGCGCAGATCTGTCGACGATCCCAATCGTAAATATGATGGAA |
| up_*mprF*_R | GTGATTCCCAAAGCGAATTATTTGTTTTTAACTTCCTGATTCATTTT |
| down_*mprF*_F | ATCAGGAAGTTAAAAACAAATAATTCGCTTTGGGAATCACTTTC |
| down_*mprF*_R | TGCAGGCATGCAAGCTTGATAAGTCACCTTCAGGGTCGTG |
